# Supplementary material for: Lineage-defined leiomyosarcoma subtypes emerge years before diagnosis and determine patient survival
Source: Nat Commun. 2021 Jul 23;12:4496. doi: 10.1038/s41467-021-24677-6 (PMC8302638; doi:10.1038/s41467-021-24677-6)
Supplement: Supplementary file 1 — Supplementary Information [file 41467_2021_24677_MOESM1_ESM.pdf]

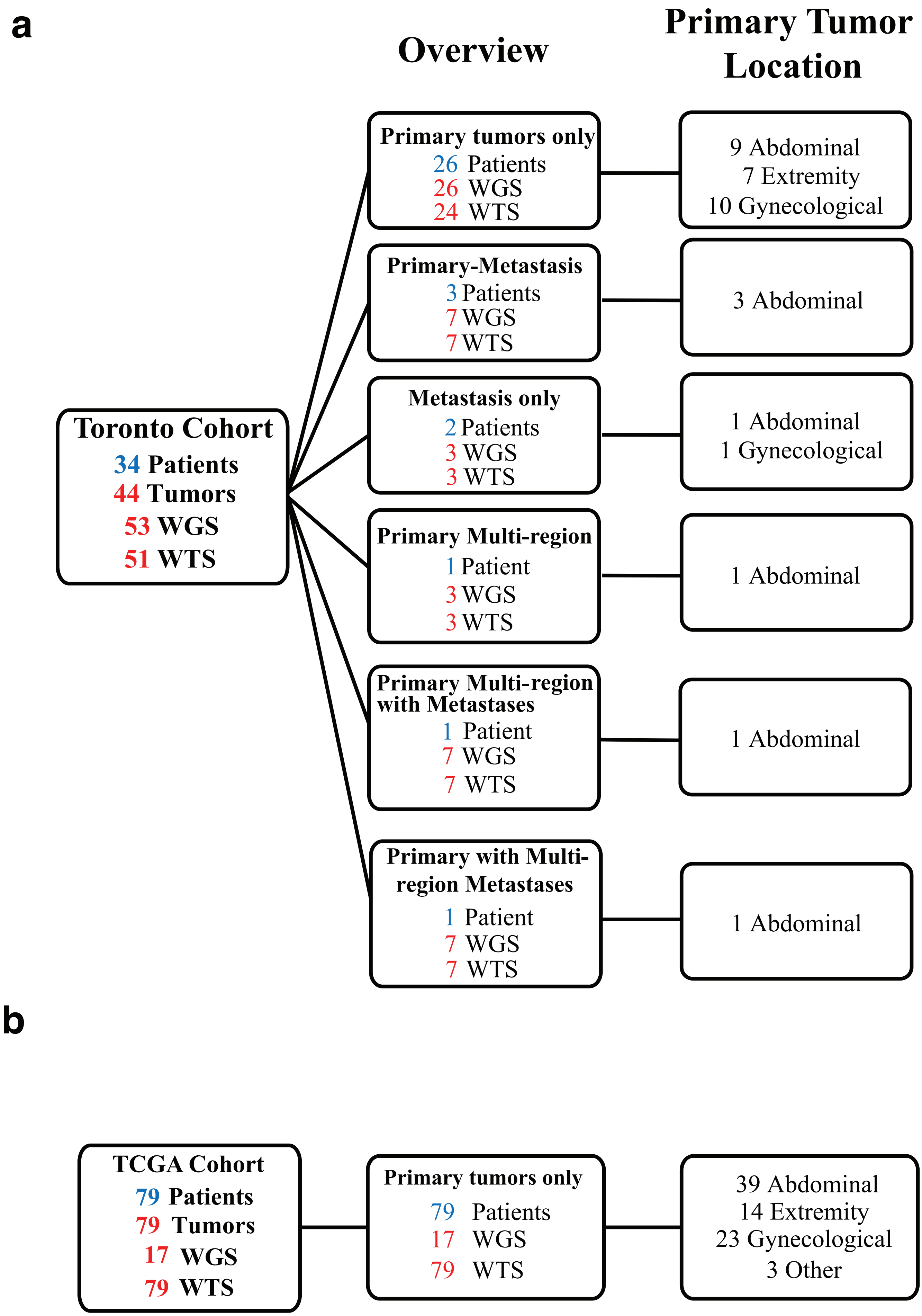


Supplementary Figure 1

**Supplementary Figure 1. Whole Genome (WGS) and Whole Transcriptome (WTS) sequencing of Leiomyosarcoma. (A)** 34 Toronto patients that were diagnosed with LMS were included in this study. 8/34 Toronto patients additionally had multi-region dissection and sequencing, metastatic relapse sequencing or both. Matched-blood or matched-normal tissue was used as a control for all cases. RNA-sequencing was performed for 51/53 Toronto samples. **(B)** Raw sequencing data from 18 LMS genomes and 80 LMS transcriptomes were obtained from The Cancer Genome Atlas (TCGA). 1 TCGA sample was removed from subsequent analysis, after detection of a *KIT* variant (indicative of gastrointestinal stromal tumor not LMS).

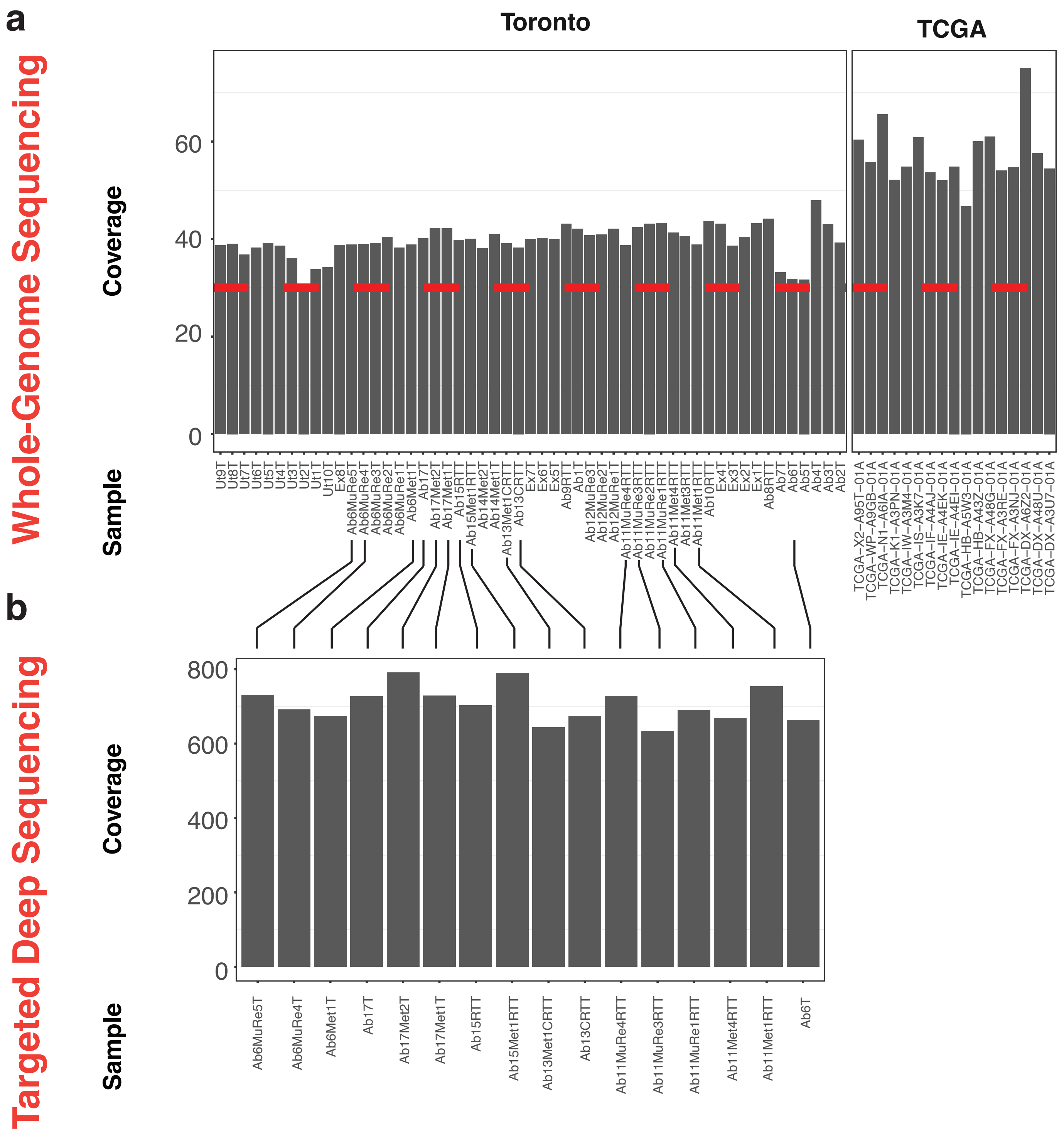


**Supplementary Figure 2. Genome Sequencing Coverage Metrics for LMS Tumors.** (A) Genome coverage was calculated using the Genome Analysis Toolkit (GATK, v.2.8.1). A minimum threshold of 30X was achieved (red dashed line), but most genomes had coverage up to 40X coverage. (B) Sixteen tumors were re-sequenced using Agilent SureSelect Technology. Multi-region and paired primary-relapse samples were prioritized. Samples were also selected based on DNA availability. Samples were sequenced to 600X-800X, (mean=706X). Full panel design details are described in Supplementary Figure 20.

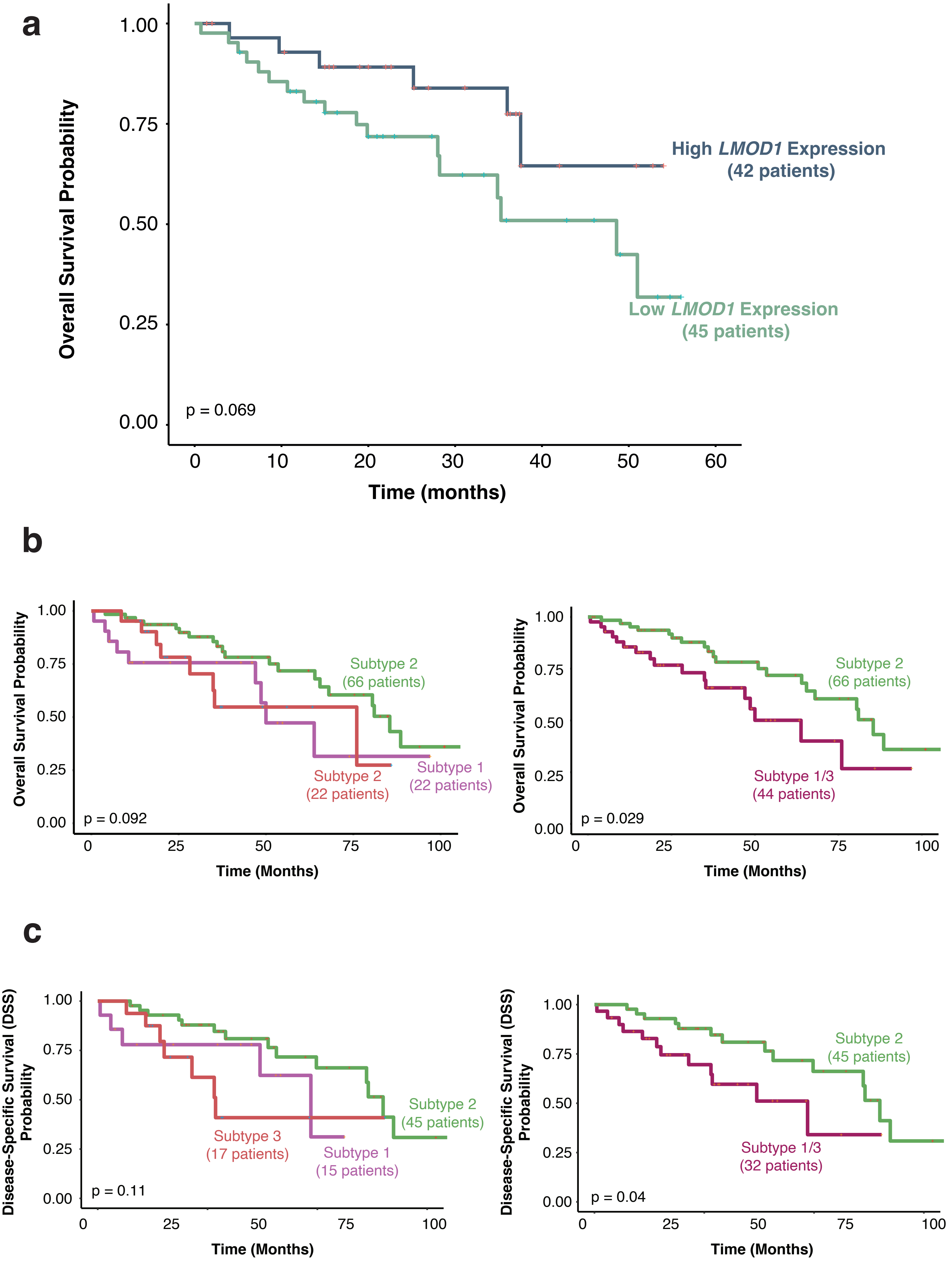


Supplementary Figure 3

**Supplementary Figure 3. Survival Differences in LMS Molecular Subtypes. (A)** Patients were divided into *LMOD1* high and *LMOD1* low groups based on their *LMOD1* expression (above/below one standard deviation of the mean). Irrespective of molecular subtype, *LMOD1* expression may be a predictor of overall survival, as low *LMOD1* expression correlated with inferior outcome. **(B)** Kaplan-Meier survival plots show that subtype 2 LMS has better overall survival than subtypes 1/3 (p=0.029, log rank test). **(C)** Kaplan-Meier survival plots show that subtype 2 LMS has better disease-specific survival than subtypes 1/3 (p=0.04, log rank test).

**a**

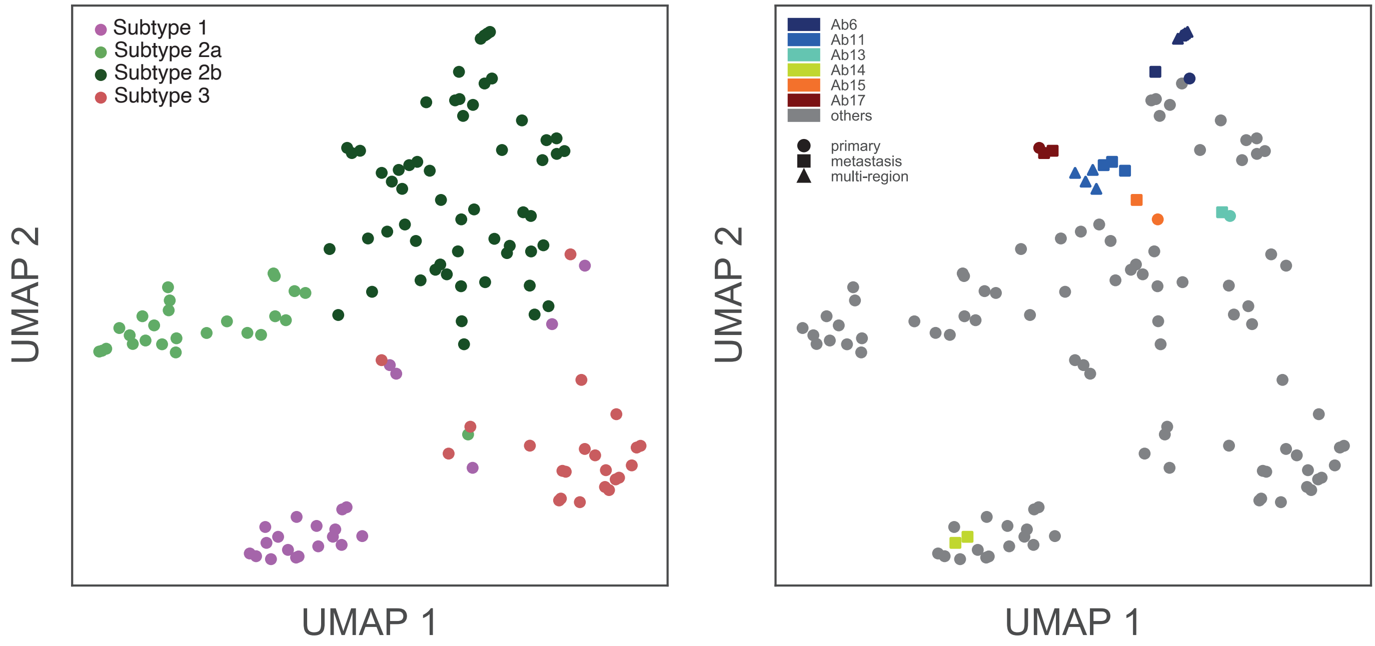


**b**

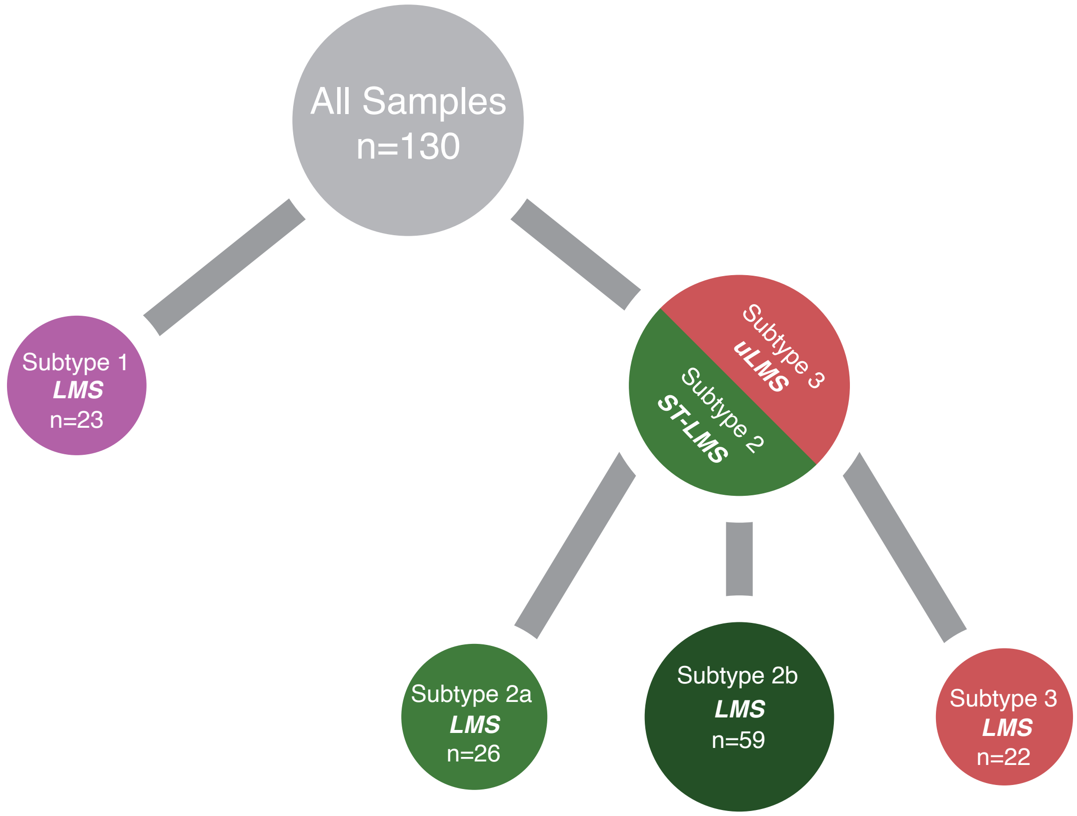


Supplementary Figure 4

**Supplementary Figure 4. UMAP of DBSCAN Hierarchical Clustering. (A)** 130 LMS transcriptomes were clustered by Density-based spatial clustering of applications with noise (DBSCAN), recapitulating clusters observed by Principal Component Analysis (left UMAP, also see Fig. 1A). Additional inclusion of 12,419 other cancers resulted in the sub-stratification of subtype 2 into subtype 2a and 2b. For Toronto patients with more than one sample (Ab6, Ab11, Ab13, Ab14, Ab15, Ab17), samples from the same patient cluster together (right UMAP). **(B)** Hierarchal distribution and visualization of uterine LMS (uLMS) and soft-tissue LMS (ST-LMS) molecular subtypes.

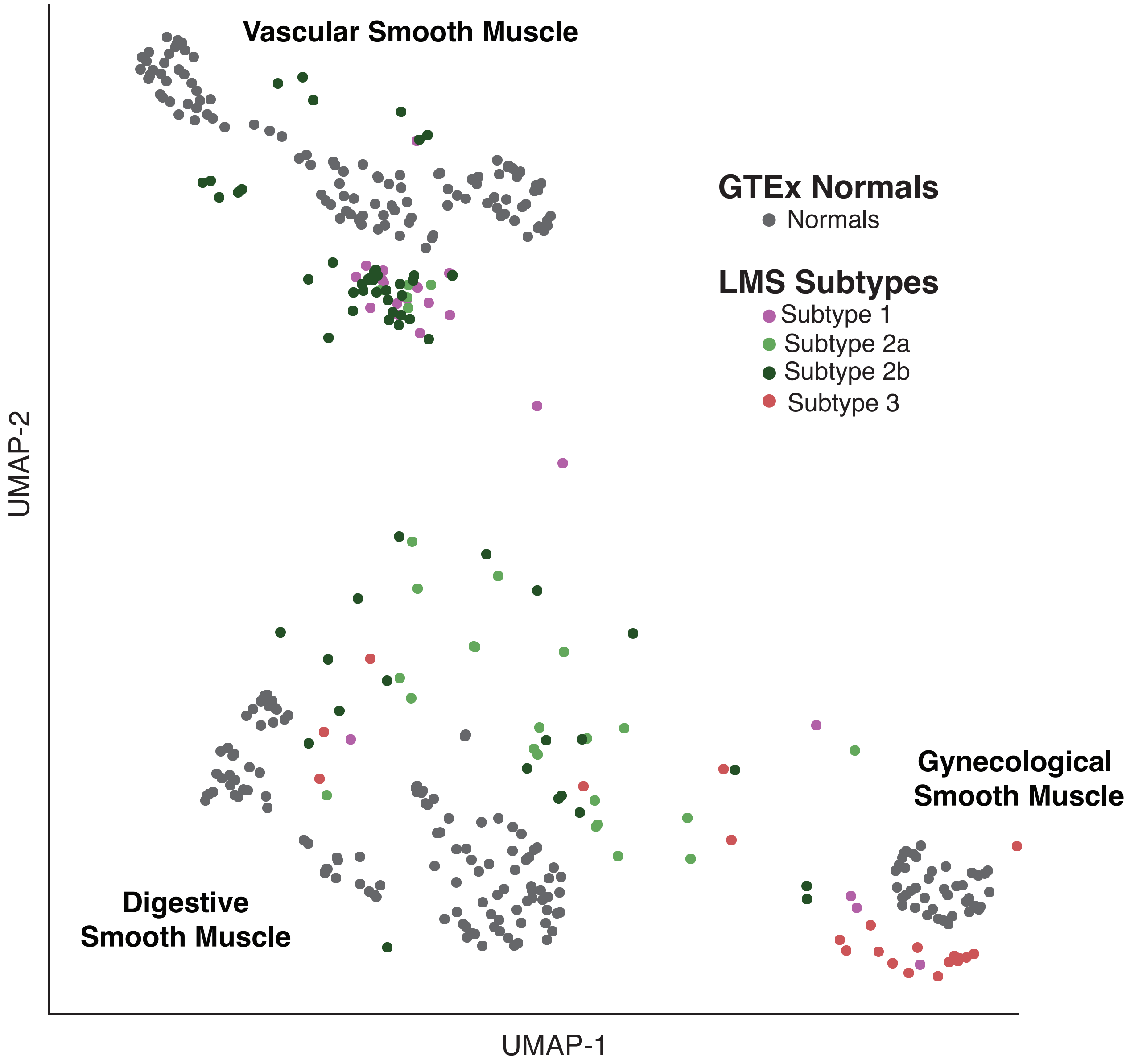


**Supplementary Figure 5. UMAP of Genotype-Tissue Expression (GTEx) Normals and LMS Cancers.** Uniform Manifold Approximation and Projection (UMAP) illustrates GTEx muscle-related normal tissues and LMS molecular subtypes clustering. LMS molecular subtypes cluster with different normal tissue of smooth muscle origin.

**a**

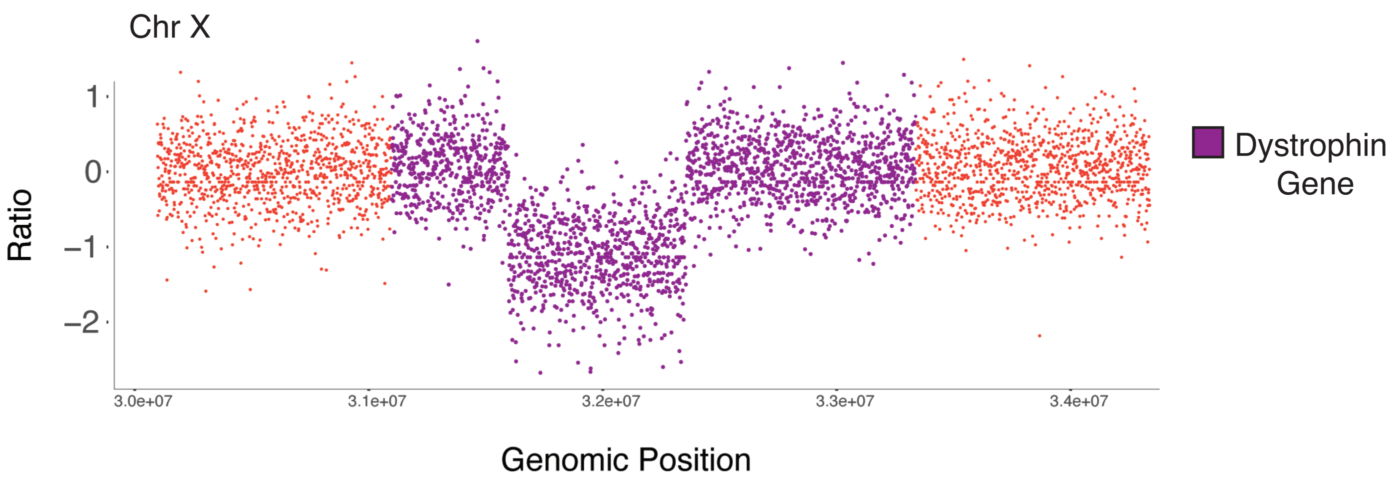


**b**

**Segmental *TP53* Loss / *MYOCD* Gain**

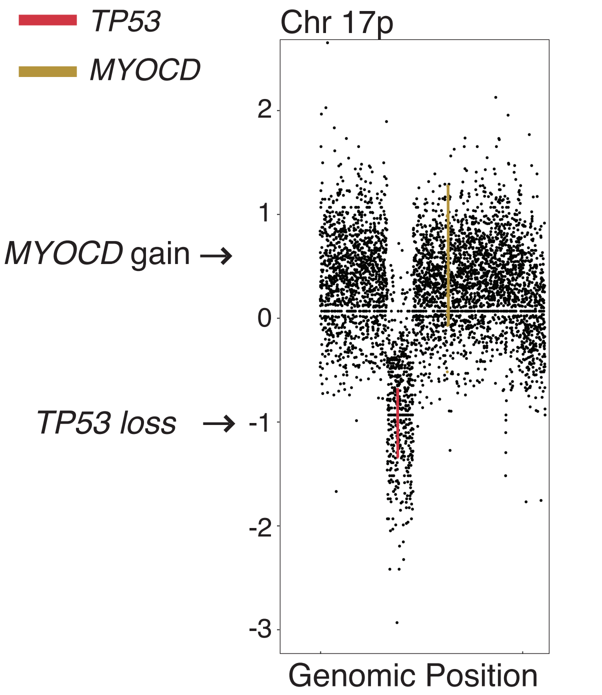


**c**

**Complex Patterns of *MYOCD* amplification**

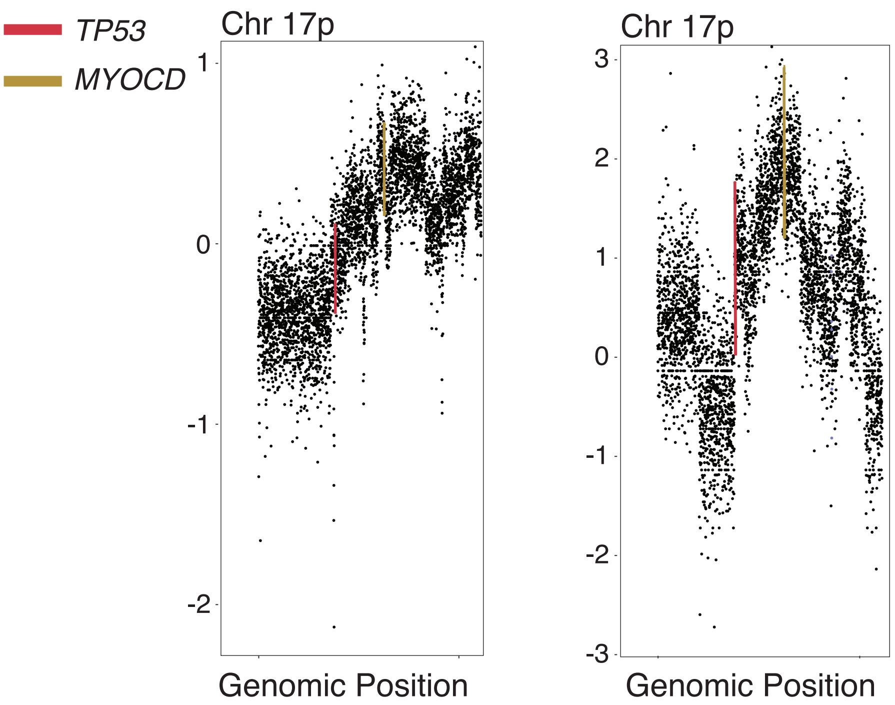


**d**

| | Subtype 1 | Subtype 2 | Subtype 3 |
|---|---|---|---|
| ***DMD* Del** | 5 | 1 | 3 |
| ***DMD* WT** | 4 | 47 | 8 |

| | Subtype 1 | Subtype 2 | Subtype 3 |
|---|---|---|---|
| ***MYOCD* Amp** | 3 | 20 | 8 |
| ***MYOCD* WT** | 6 | 28 | 3 |

**Total: 68 Genomes**

Supplementary Figure 6

**Supplementary Figure 6. Genomic Alterations in Smooth Muscle Genes. (A)** Copy number plot of a representative LMS sample with an intragenic dystrophin (*DMD*) deletion. **(B)** Copy number plot shows a segmental 17p loss (*TP53*), accompanied by a neighbouring 17p gain (*MYOCD*) in a representative sample. **(C)** Copy number plot illustrates two indicative samples illustrating complex patterns of copy-number alterations resulting in *MYOCD* amplification. **(D)** Summary table of *DMD* deletions (*DMD* Del) and *MYOCD* amplifications (*MYOCD* amp) detected by WGS. *DMD* deletions occur predominantly in subtypes 1/3, while *MYOCD* amplifications occur preferentially in subtypes 2/3. "Wildtype" (WT) refers to samples where no alteration in *DMD* or *MYOCD* was detected.

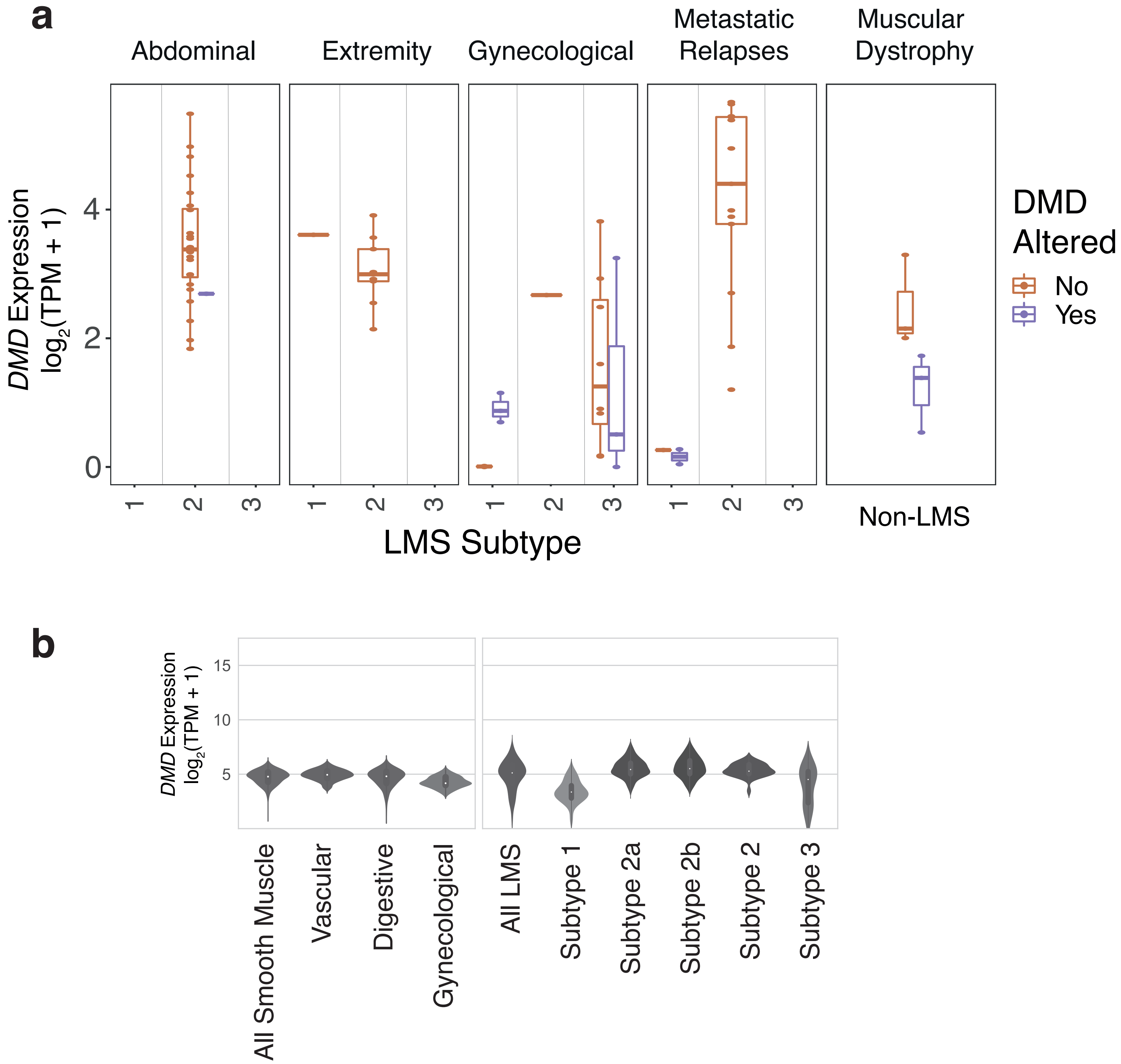


**Supplementary Figure 7. Dystrophin Expression in LMS.** (A) Boxplot illustrates dystrophin expression (in transcripts per million, TPM) for LMS cancers with paired genome and RNA sequencing. Samples are categorized by subtype (subtype 1 = 9, subtype 2 = 48, subtype 3 = 11) and anatomical location (abdominal = 25, extremity = 10, gynecological = 17, metastatic relapses = 16). 6 muscular dystrophy cases were included. The boxes represent the 25th and 75th percentile (bottom and top of box), and median value (horizontal band). The whiskers indicate the variability outside the upper and lower quartiles. Most dystrophin deletions arise in subtype 1 and 3 gynecological LMS. Gynecological LMS have lower dystrophin expression regardless of deletion status. For subtype 1 metastatic relapses, two relapses from the same patient harbor the same *DMD* deletion. The other specimen does not harbor a *DMD* deletion and is derived from a gynecological primary tumor (not sequenced). Lower *DMD* expression is observed in muscular dystrophy patients with a *DMD* alteration (compared to alterations in related genes). (B) Violin plots demonstrate dystrophin expression across normal GTEx muscle tissues (vascular = 110, digestive = 119 and gynecological = 42) and LMS molecular subtypes (subtype 1 = 23, subtype 2 = 85, subtype 3 = 22).

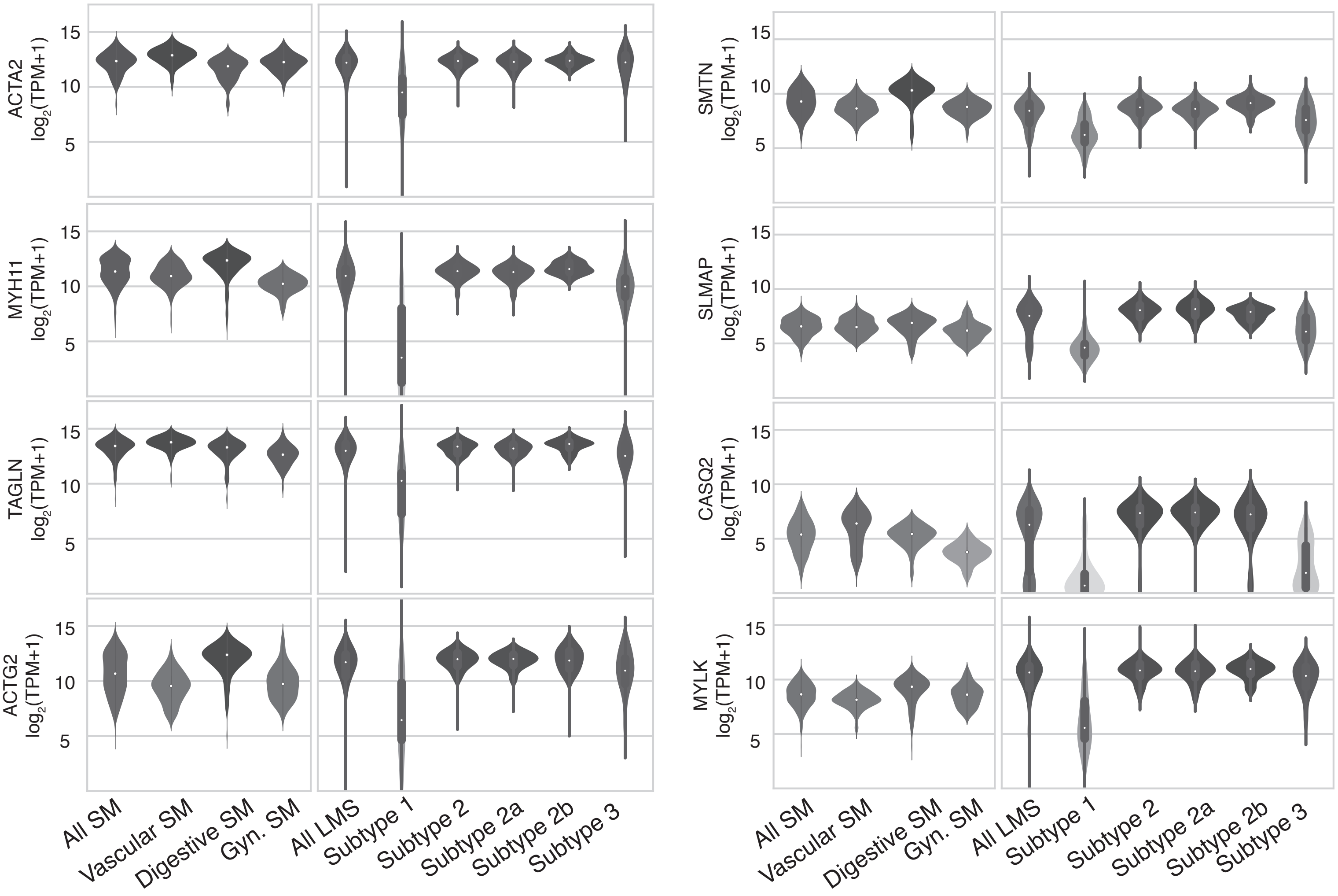


**Supplementary Figure 8. Smooth Muscle (SM) Marker Expression (Additional Genes).** Boxplots represent the expression (in transcripts per million, TPM), for SM genes. The boxes represent the 25th and 75th percentile (bottom and top of box), and median value (horizontal band). The whiskers indicate the variability outside the upper and lower quartiles. These 8 muscle genes were reported in Beck et al. 2014 as the defining features of LMS molecular subtype 2 (Beck et al.'s subgroup 1). These smooth muscle markers are under-expressed in LMS subtype 1 (n=23), to differing degrees, relative to subtypes 2 (n=85) or 3 (n=22). SM expression in vascular (n=110), digestive (n=119) and gynecological (Gyn., n=42) normal tissues are also shown.

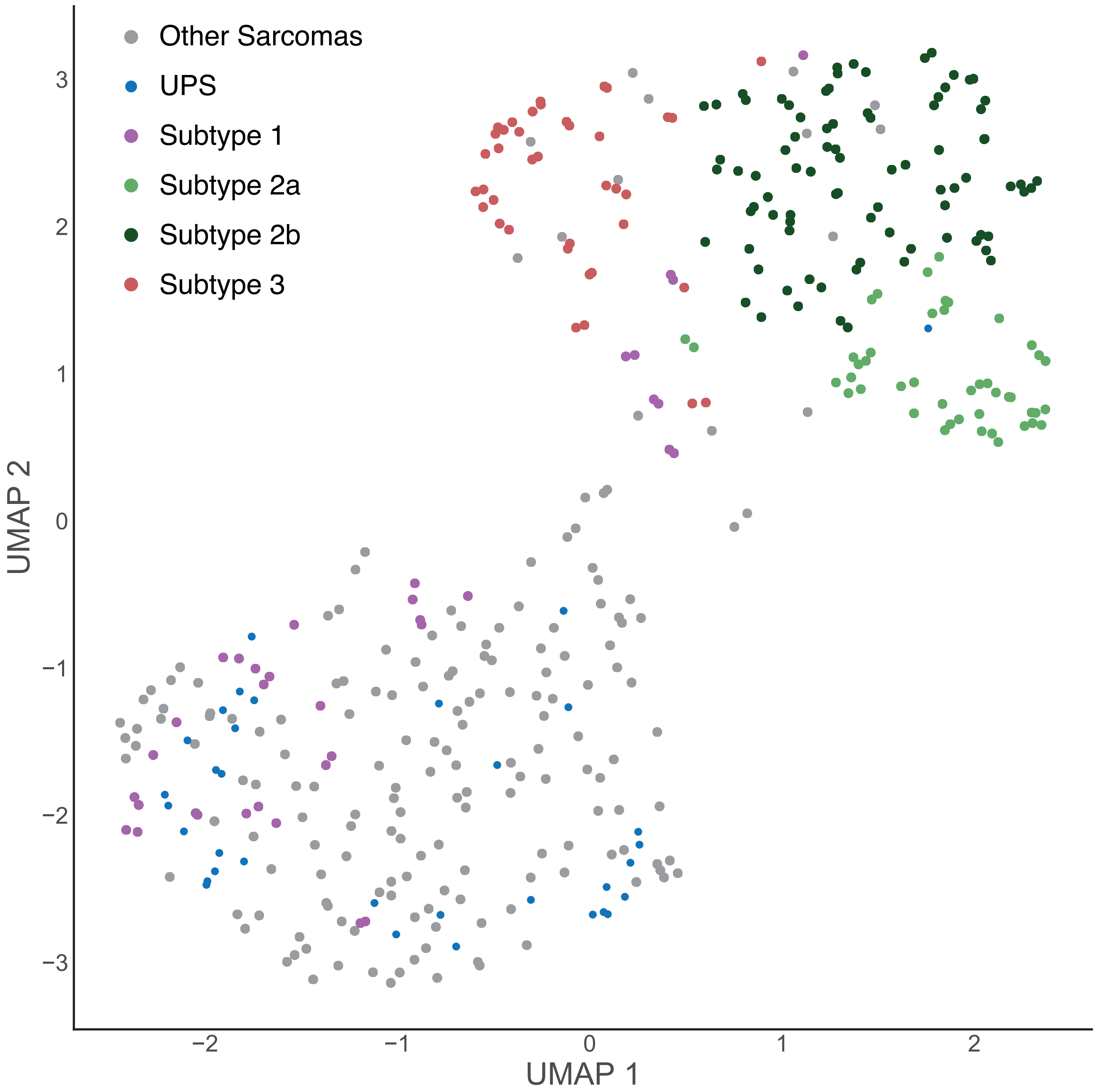


**Supplementary Figure 9. Subtype 1 LMS clusters with Undifferentiated Pleiomorphic Sarcoma (UPS).** Uniform Manifold Approximation and Projection (UMAP) demonstrates that LMS subtype 1 cancers cluster with UPS tumors obtained from The Cancer Genome Atlas (TCGA).

**a**

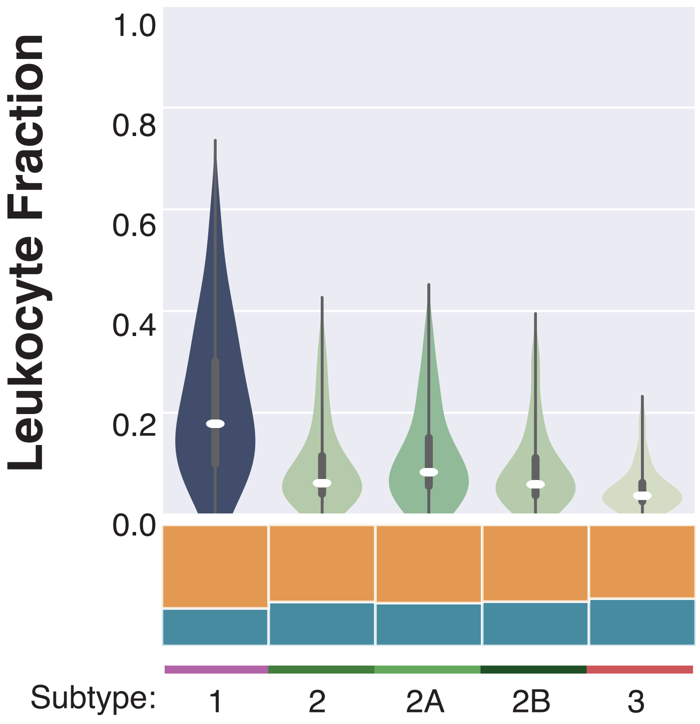


**b**

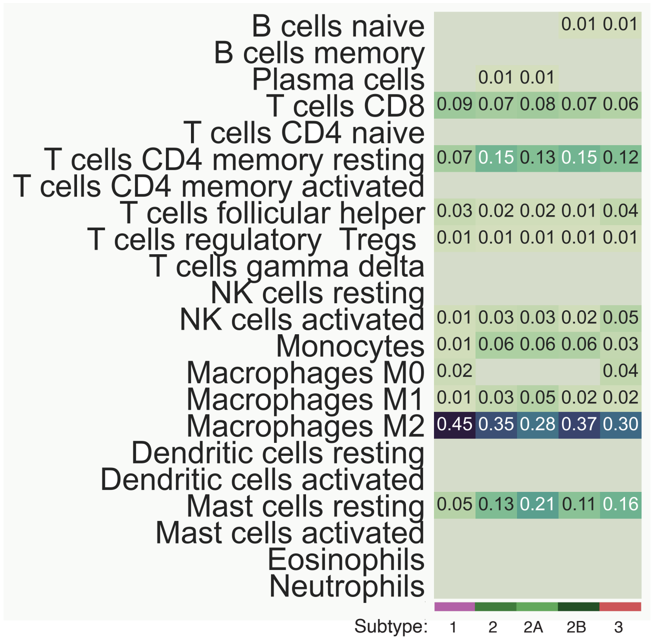


**c**

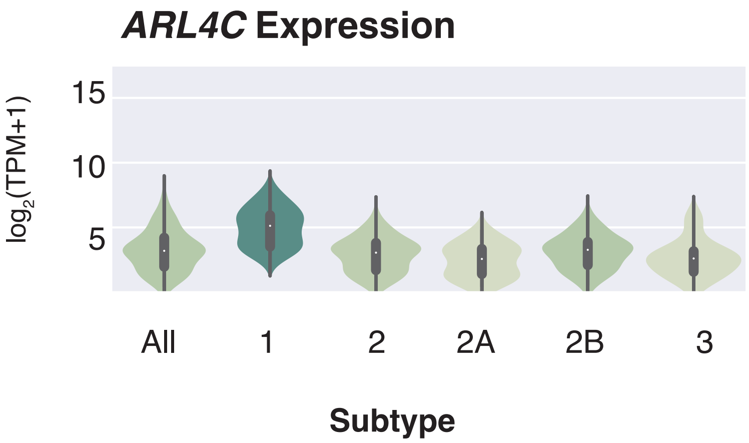


Supplementary Figure 10

**Supplementary Figure 10. Higher Immune Infiltration in LMS Subtypes. (A)** Boxplot demonstrates leukocyte proportions in LMS subtypes. The boxes represent the 25th and 75th percentile (bottom and top of box), and median value (horizontal band). The whiskers indicate the variability outside the upper and lower quartiles. A higher leukocyte fraction is observed in Subtype 1 (n=16) than Subtypes 2 (n=46) or 3 (n=17). Only TCGA samples were analyzed. Orange bars represent myelocytes, whereas blue bars represent lymphocytes. **(B)** Heatmap represents lymphocyte and monocyte breakdown of leukocyte content reveals higher M2 macrophage content in LMS, with an enrichment in subtype 1 tumors (darker blue/higher number refers to higher proportion). Only TCGA samples were analyzed. **(C)** *ARL4C* expression is higher in Subtype 1 (n=23) than in subtypes 2 (n=85) or 3 (n=22).

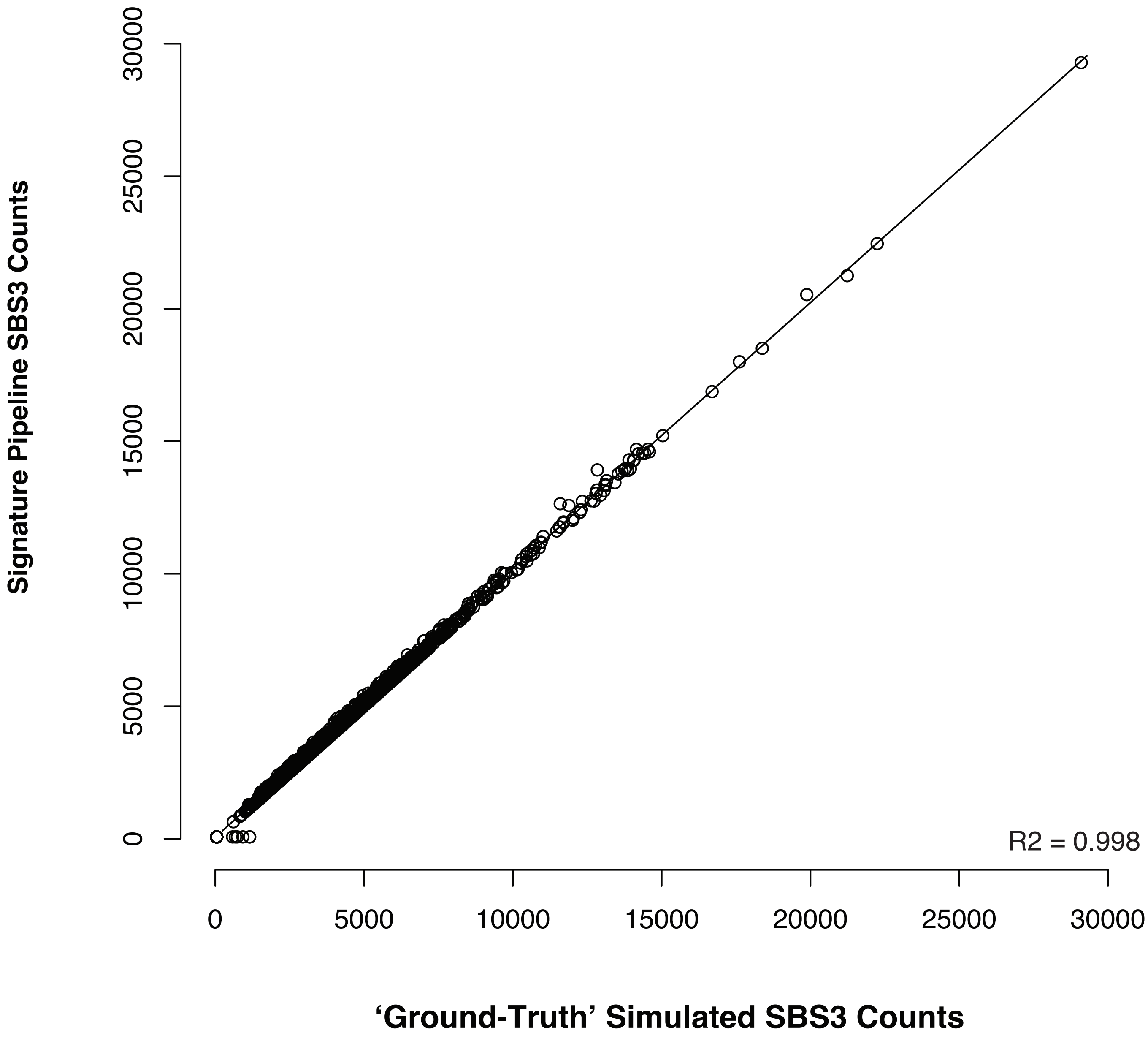


**Supplementary Figure 11. Single Base Substitution (SBS) 3 Validation: Pan-Cancer Analysis of Whole Genomes (PCAWG) Ground Truth.** A ground-truth positive control dataset was obtained from PCAWG Network where SBS3, SBS5 and SBS40 were simulated in 1000 samples. The ground-truth dataset was compared to signature output from this study. The two-sided Pearson correlation coefficient was >0.99 indicating a strong correlation and sufficient power to discriminate SBS3 from SBS5 and SBS40.

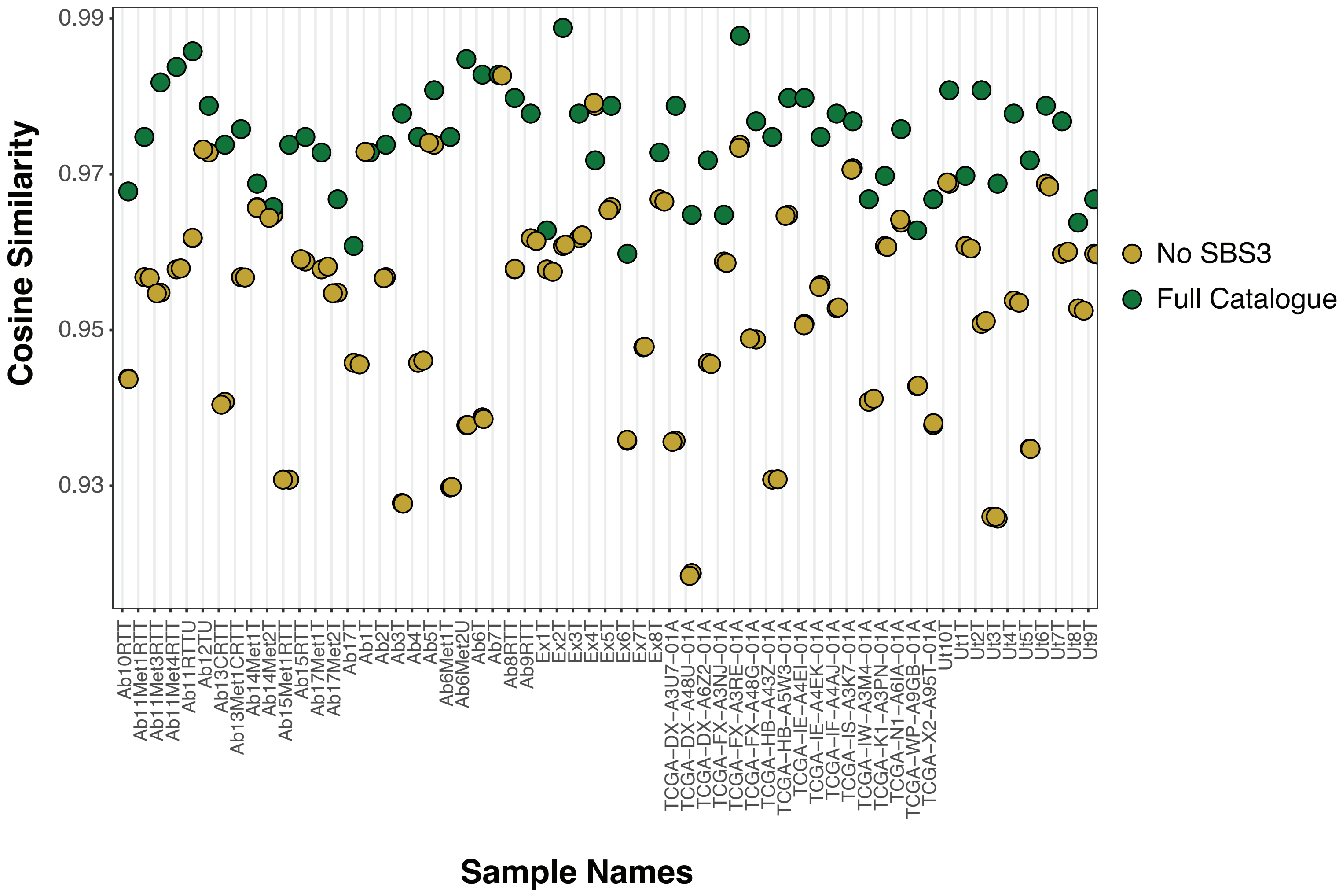


**Supplementary Figure S12. Single Base Substitution (SBS) 3 Validation: Removing SBS3 from Decomposition.** First, signatures were *de novo* extracted and decomposed using the full catalogue of available COSMIC signatures. The cosine similarities between the reconstructed signatures and COSMIC signatures are plotted in green. Removal of SBS3 from the catalogue of signatures available for signature decomposition results in a decrease in cosine similarity (plotted in yellow).

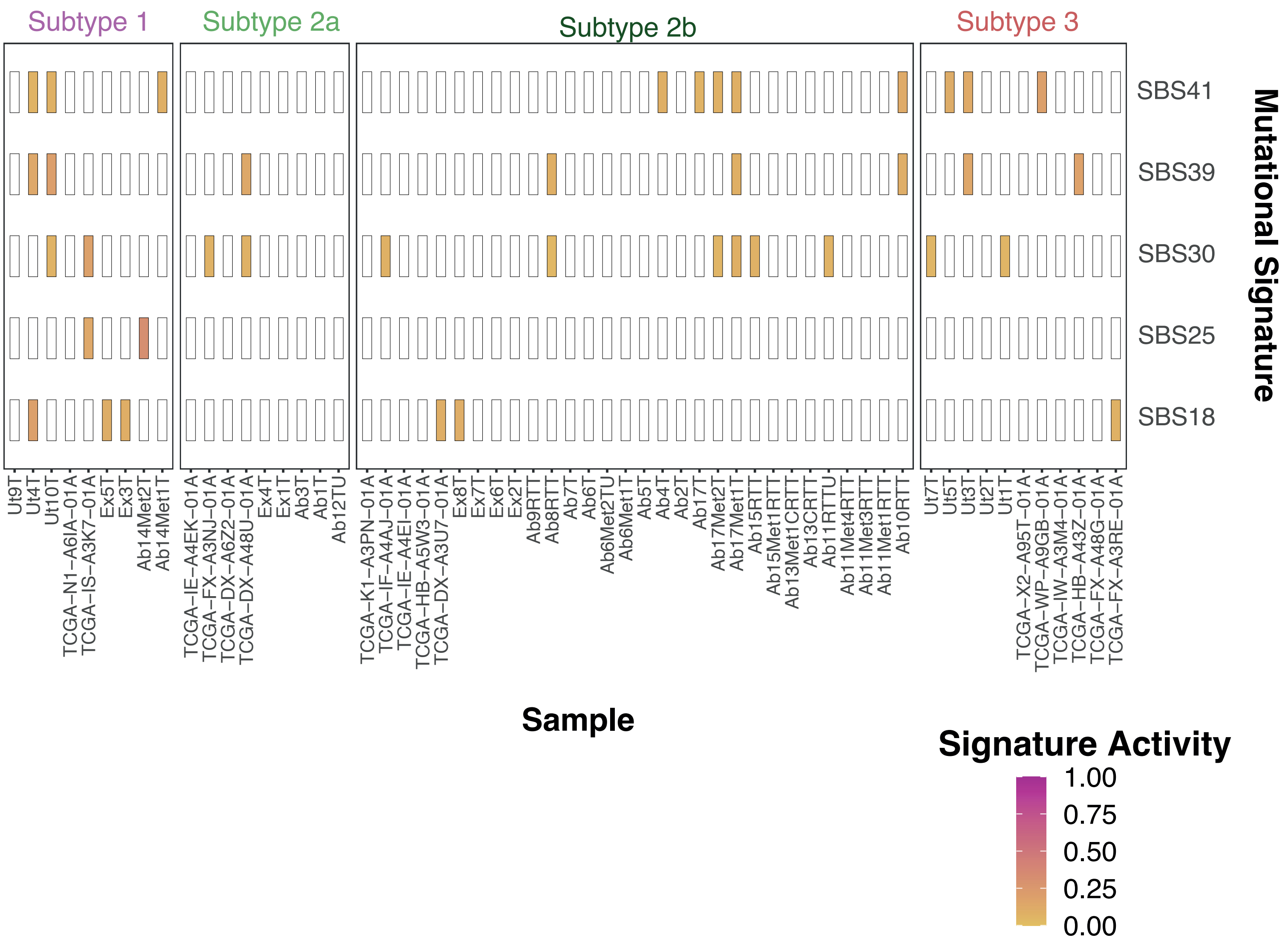


**Supplementary Figure 13. Infrequent Substitution Signatures.** The heatmap illustrates the signature activity of non-negative matrix factorization (NMF)-extracted substitution signatures present in less than 5% of samples. In Figure 2A, these are summarized as ‘Other’. Source data are provided as a Source Data file.

a

# Unknown Indel Signatures

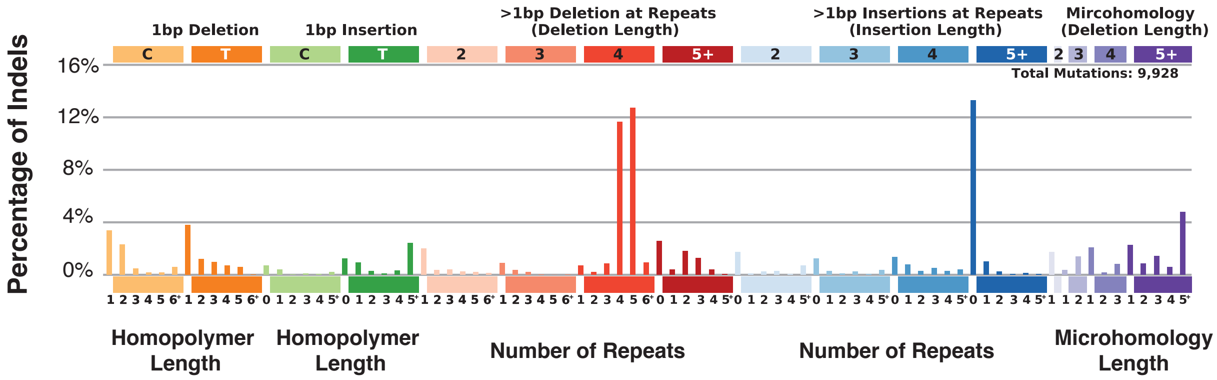


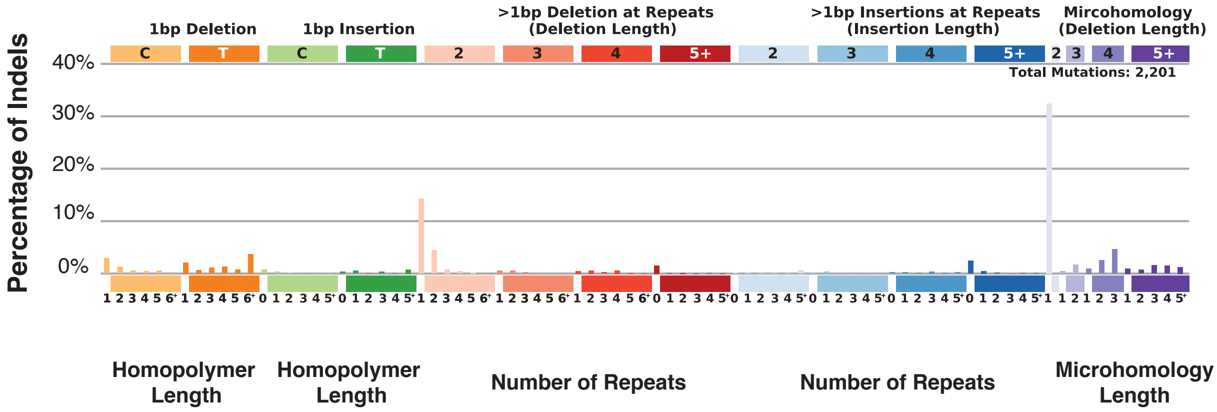


b

# Unknown Double-Nucleotide Signatures

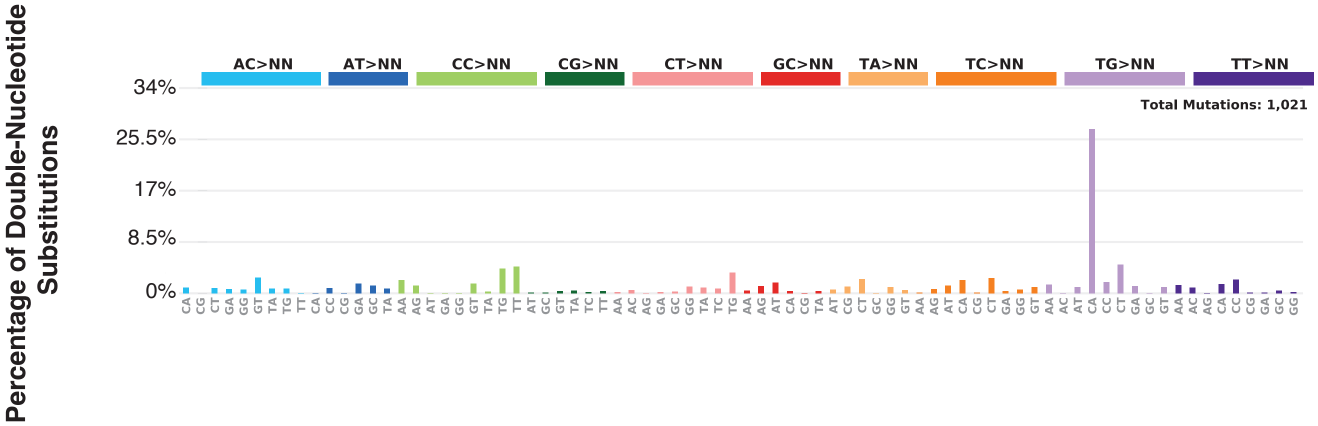

**Supplementary Figure 14. Unidentified Signatures in LMS.** Non-negative matrix factorization (NMF)-extracted signatures revealed 3 signatures (two indel and one double-nucleotide substitution) that were not identified in COSMIC, to date. These may represent novel signatures in LMS.

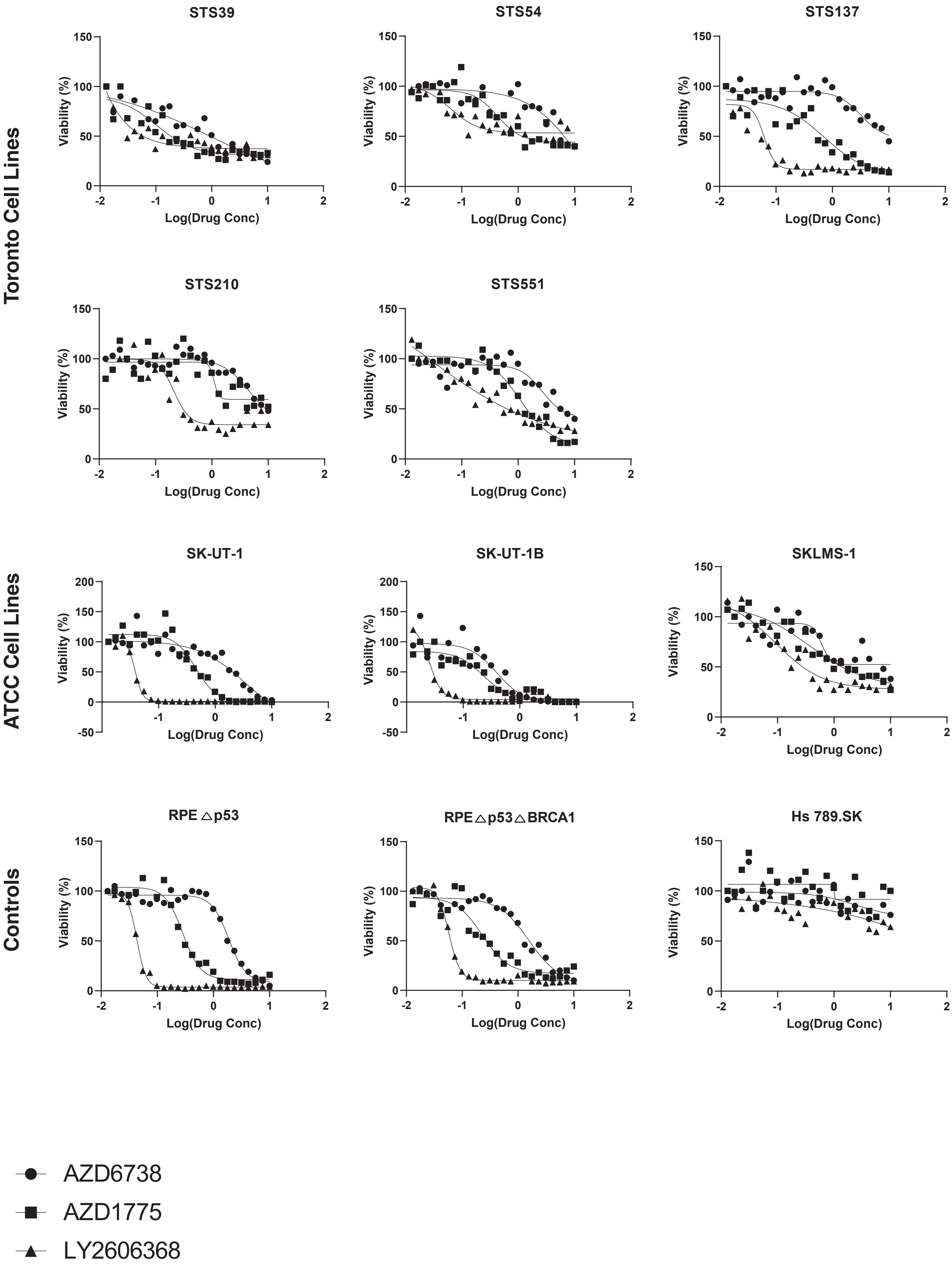


Supplementary Figure 15

**Supplementary Figure 15. LMS Cell Lines are Responsive to DNA Response Inhibitors (DDRi).** LMS cell lines and controls were treated with inhibitors for CHK1 (LY2606368), ATR (AZD6738), WEE1(AZD1775) to create dose response curves using 24 distinct concentrations (0.013-10μM) with biological triplicates. Hs 789.Sk represents a non-transformed fibroblast cell line. Doses were randomized in a scattered manner across the plates to minimize positional artifacts. The $EC_{50}$ values were calculated in Prism using a sigmoidal curve fit with three parameters. Vehicle treated cells were normalized to 100%. Source data are provided as a Source Data file.

**a**

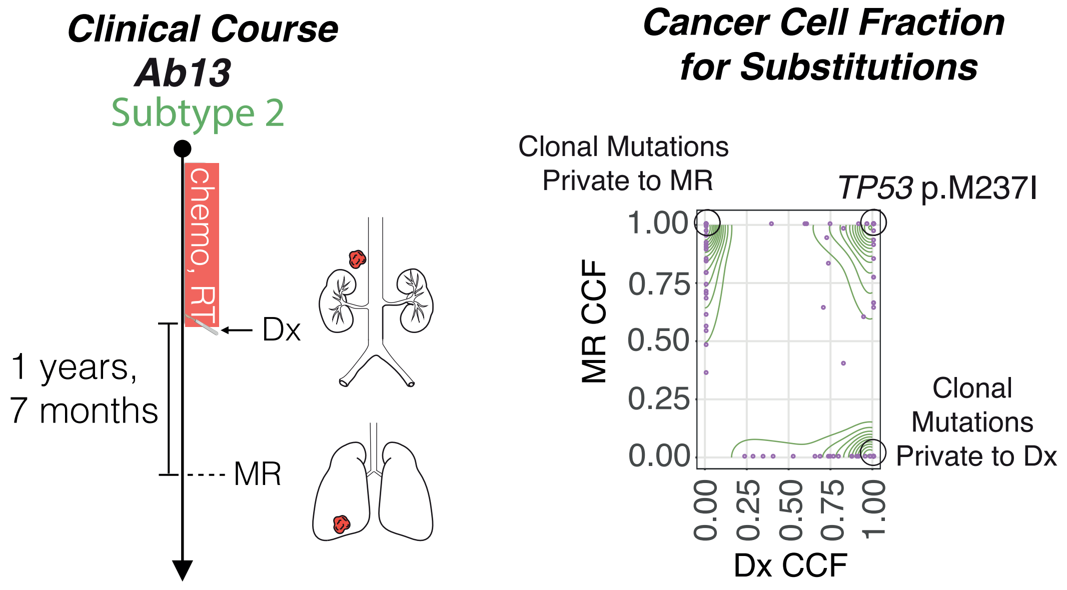


**b**

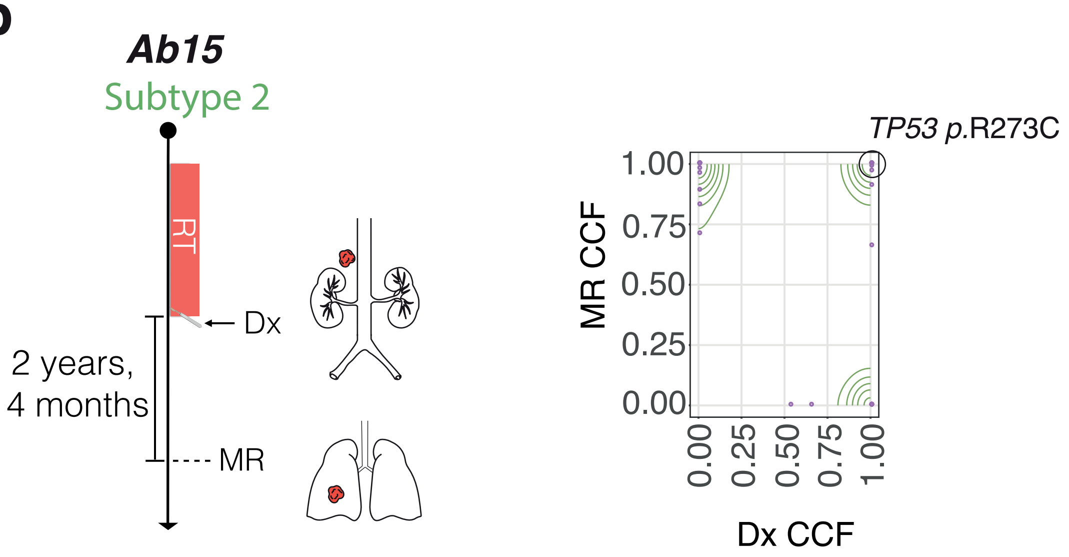


**c**

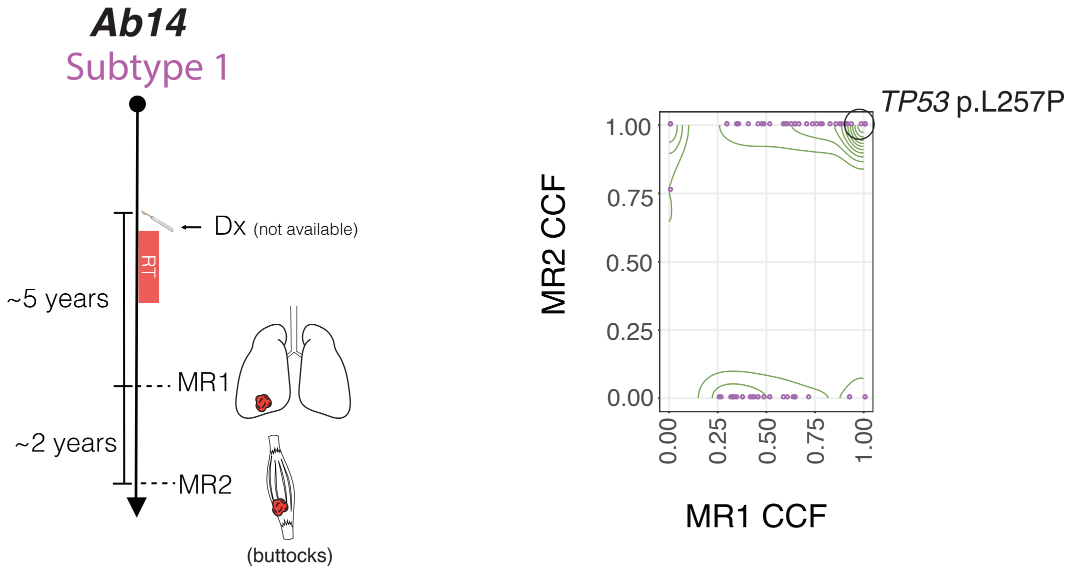


Supplementary Figure 16

**Supplementary Figure 16. Bulk Sequencing of Primary and Metastatic Relapse Pairs (n=2 samples per patient).** The clinical courses of two primary-relapse pairs (Ab13 and Ab15) and one relapse-relapse pair are shown (left column). All patients were treated with either chemotherapy (chemo) or radiation therapy (RT). For Ab14, only the metastatic relapses (MR1 and MR2) were genome and RNA-sequenced, as the diagnostic tumor (Dx) was not available. Pathogenic *TP53* substitutions with clonal cancer cell fractions (CCFs) are present in all samples, thus supporting their obligate nature in LMS.

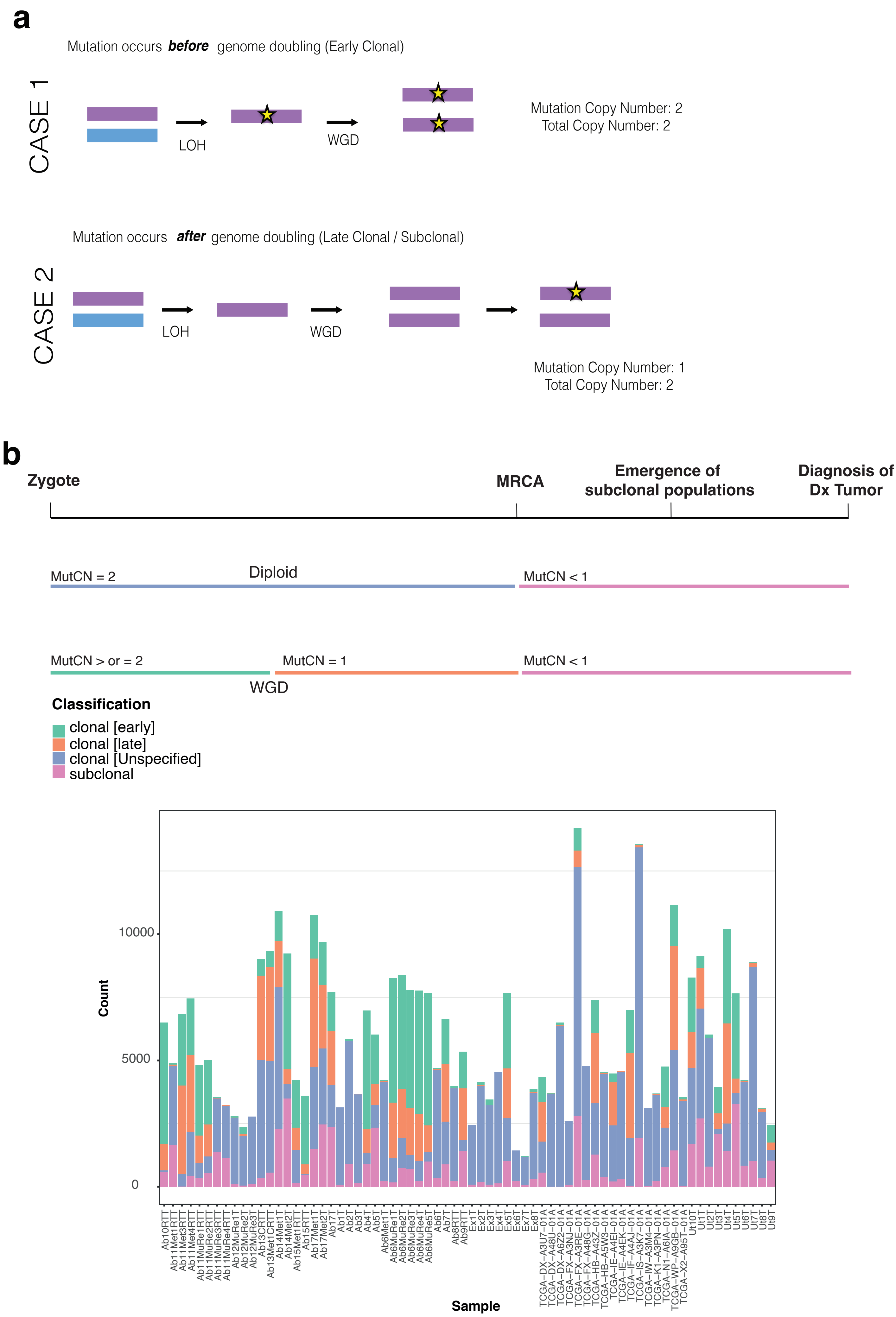


Supplementary Figure 17

**Supplementary Figure 17. Timing point mutations in LMS.** Using informative regions of the genome, point mutations can be timed relative to DNA amplifications. **(A)** In case 1, there is an initial loss-of-heterozygosity (LOH) event, followed by a pathogenic variant. Subsequent whole-genome duplication (WGD) results in co-amplification of the mutated allele. In this scenario, the mutation arises early (prior to DNA amplification). In case 2, similar LOH arises, however the mutation does not arise until after the amplification event. In this scenario, the mutation is a late event. **(B)** Using MutationTimeR, somatic mutations can be timed relative to clonal and subclonal copy number states. Variants are classified and timed given their copy number states and mutation copy number (MutCN) (see methods). In samples with chromosomal amplifications, the majority (63%) of point mutations are later events, arising after focal amplifications. 37% of mutations arise early, before these amplifications. The remaining mutations occur after the most recent common ancestor (MRCA) and before the diagnostic tumor (Dx). The most common pathogenic COSMIC cancer gene variants that arise early are *TP53.*

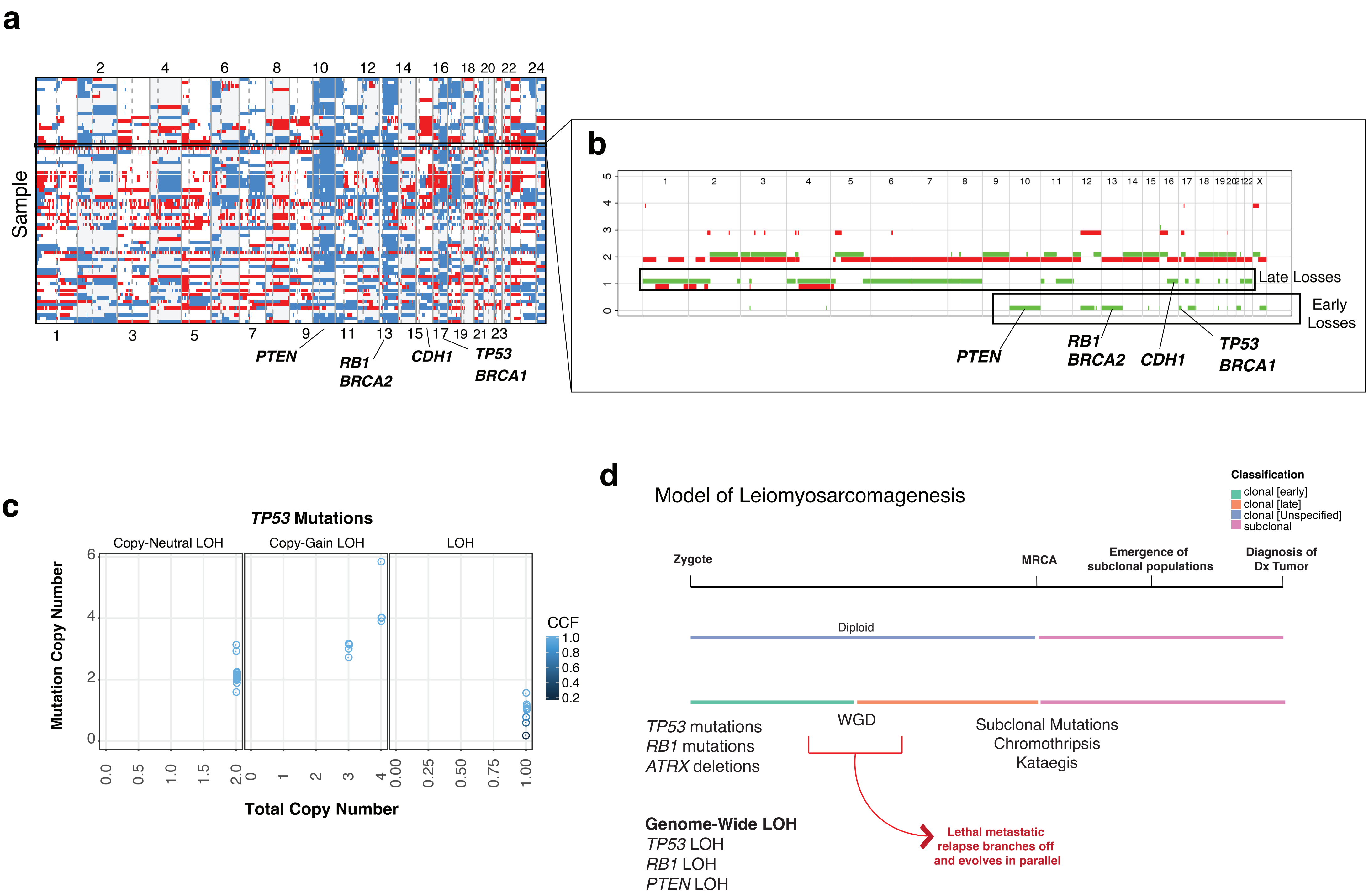


Supplementary Figure 18

**Supplementary Figure 18. Timing Copy Number Aberrations and *TP53* in LMS.** **(A)** Genome-wide copy number illustrates recurrent chromosomal losses of chromosomes 10, 13, 16, and 17. **(B)** Allele-specific copy-number in a representative sample shows that the aforementioned copy number losses are early events arising before genome doubling. **(C)** Dot plots show mutation copy number and total copy number for the most recurrent alteration in LMS, *TP53* variants. In regions of copy-neutral loss-of-heterozygosity (LOH) or copy-gain LOH (one copy loss, the other copy is amplified more than once), the *TP53* mutation copy number equals the total copy number and has a high cancer cell fraction (CCF) (see Supplementary Figure 17A). **(D)** Model for Leiomyosarcomagenesis: in an early mesenchymal progenitor, a pathogenic *TP53* is obtained. *RB1* alterations and *ATRX* deletions may also occur. These events are accompanied by genome-wide copy number losses in known tumor suppressor genes such as *TP53*, *RB1* and *PTEN*. Whole genome duplication (WGD) occurs in ~40-50% of cases. WGD is an early-mid evolutionary event, frequently common to all regions of a tumor (in cases of multi-region sequencing) or both the primary and metastatic relapse. In other cases, WGD is unique to the primary or metastatic relapse. Based on clock-like mutagenesis analysis, LMS cancers diverge 10-30 years pre-diagnosis, suggesting the most recent common ancestor (MRCA) must precede the diagnostic tumor (Dx) by many years. Kataegis and chromothripsis are mid-late events in LMS.

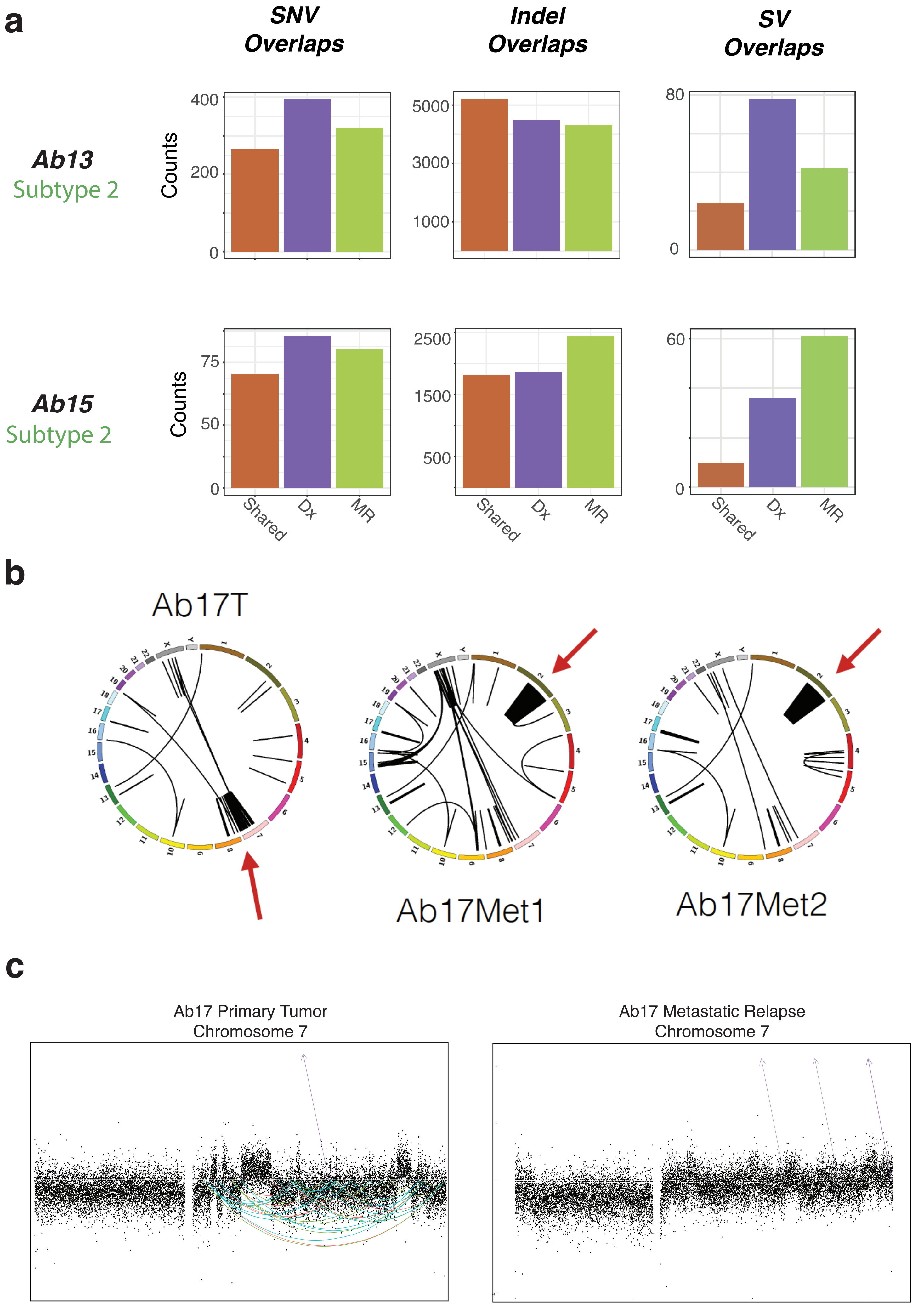


Supplementary Figure 19

**Supplementary Figure 19. Clonal Evolution in LMS. (A)** Single-nucleotide variant (SNV), indel and structural variant (SV) overlap of the bulk sequencing of primary-metastatic relapse pairs also shows the high number of unique variants present in each tumor, suggesting parallel evolution between the diagnostic sample (Dx) and the later metastatic relapse (MR). **(B)** Circos plots illustrate differing structural variation between the diagnostic tumor (Ab17T), and the two metastatic relapses (MR1=Ab17Met1 and MR2=Ab17Met2). See Fig 3A for clinical course. Two separate chromothriptic events occur between the primary and relapses. **(C)** Copy number profiles of chromosome 7 further support a chromothriptic event that is unique to the primary tumor and not observed in the metastatic relapse.

A

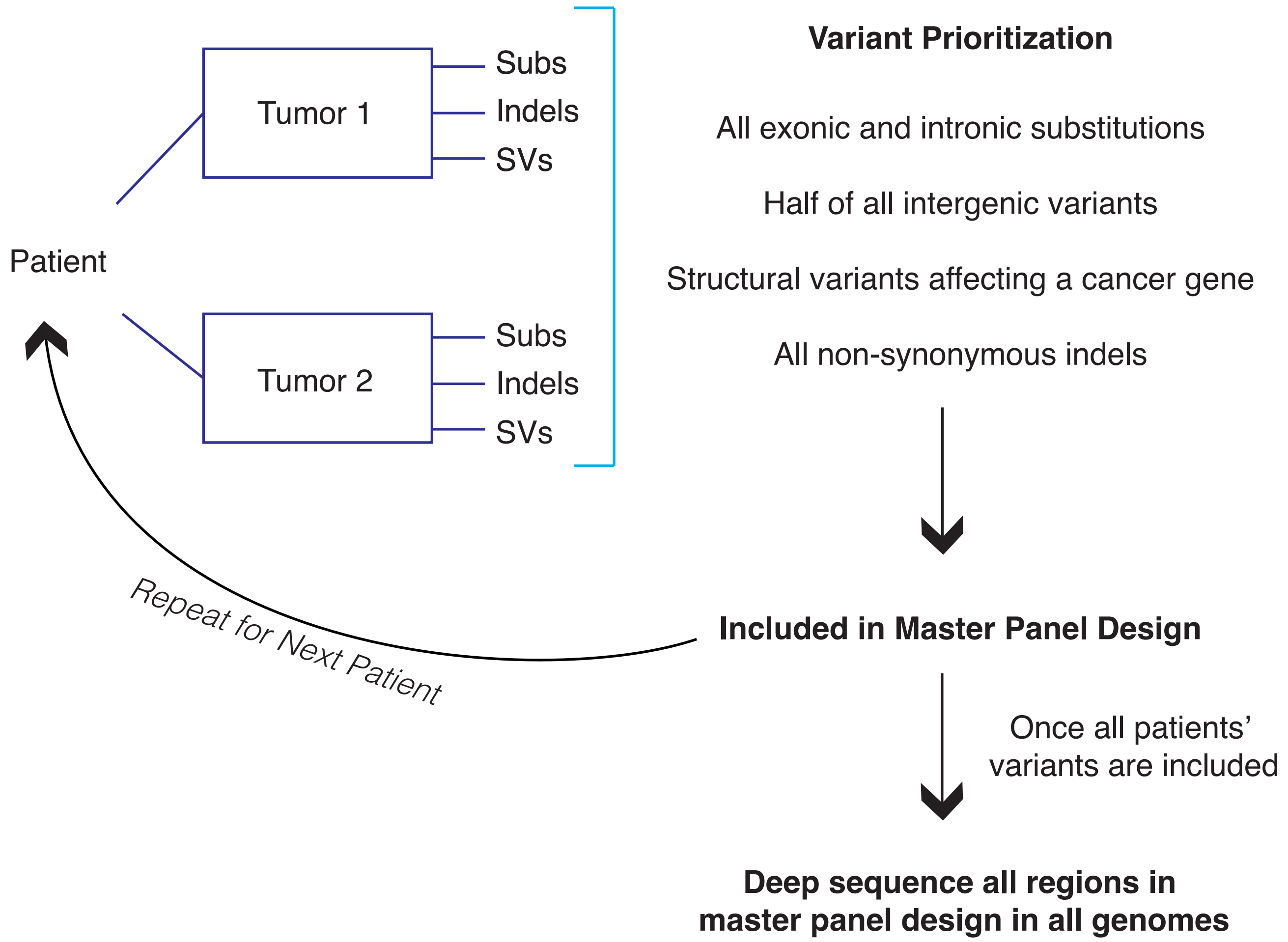


B

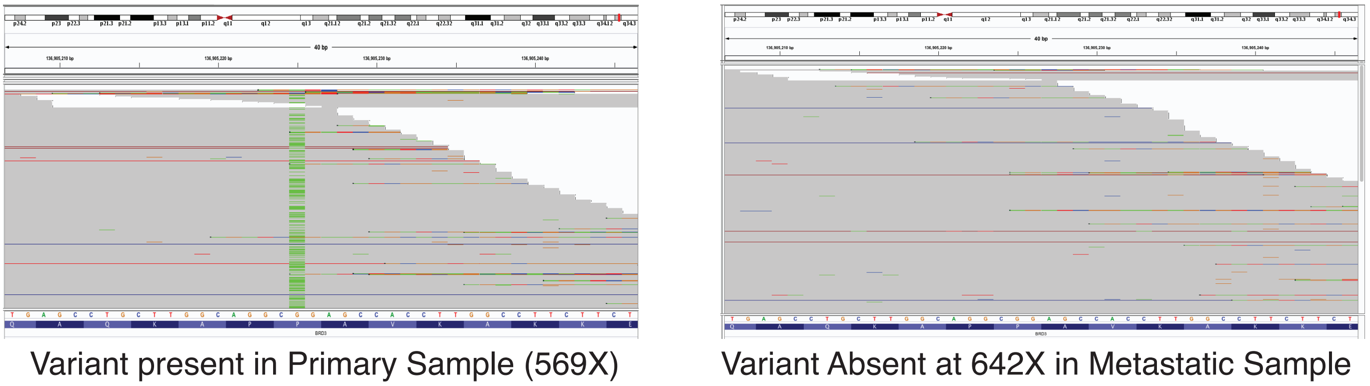


**Supplementary Figure 20. Deep, Targeted Sequencing of LMS Tumors. (A)** The flow diagram illustrates the design approach of the targeted panel for deep sequencing of LMS genomes. For any given patient with multi-region or paired primary-relapsed tumors, all substitutions (subs), insertion-deletions (indels), and structural variants (SVs) are considered. Over 75% of substitutions, all non-synonymous indels, and SVs with breakpoints within cancer genes were included in the design for any given genome. Once pooled from sixteen samples, all regions were sequenced in every patient to a mean depth of 706X. **(B)** IGV screenshots show a representative example of a substitution variant present in a diagnostic tumor that is absent in the paired metastatic sample, even at 642X sequencing depth at that position.

## Tree From Figure 3A

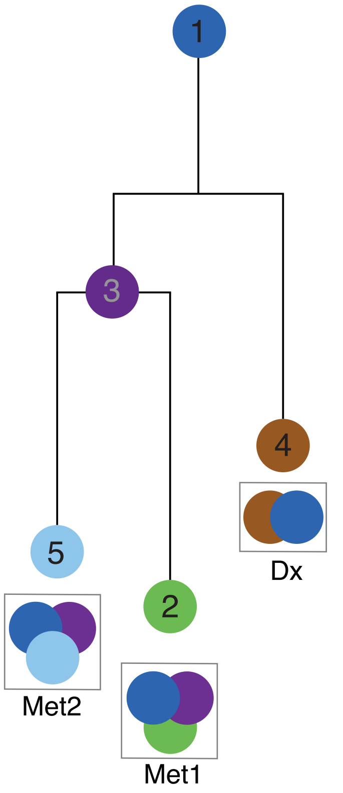


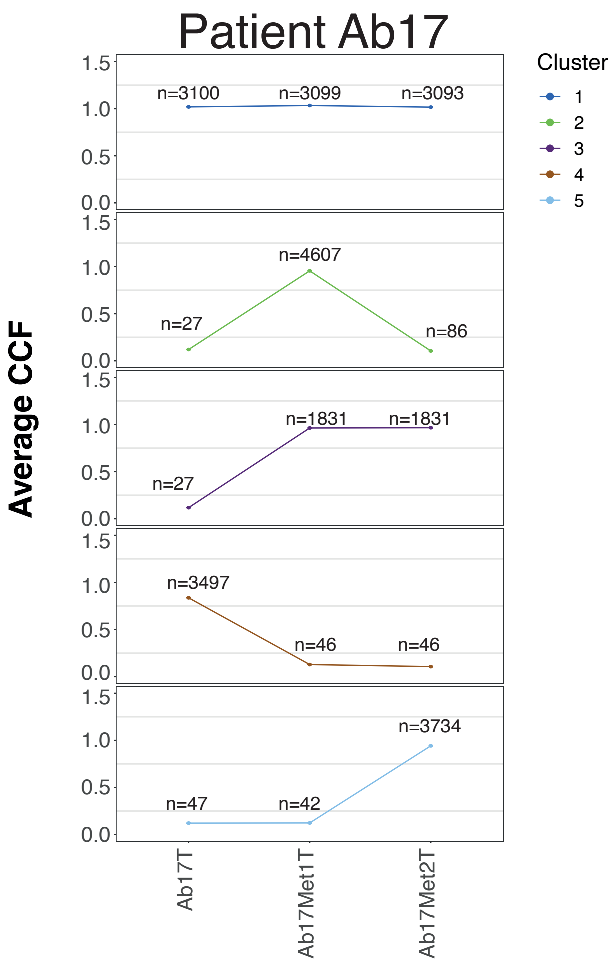


## Tree From Figure 3B

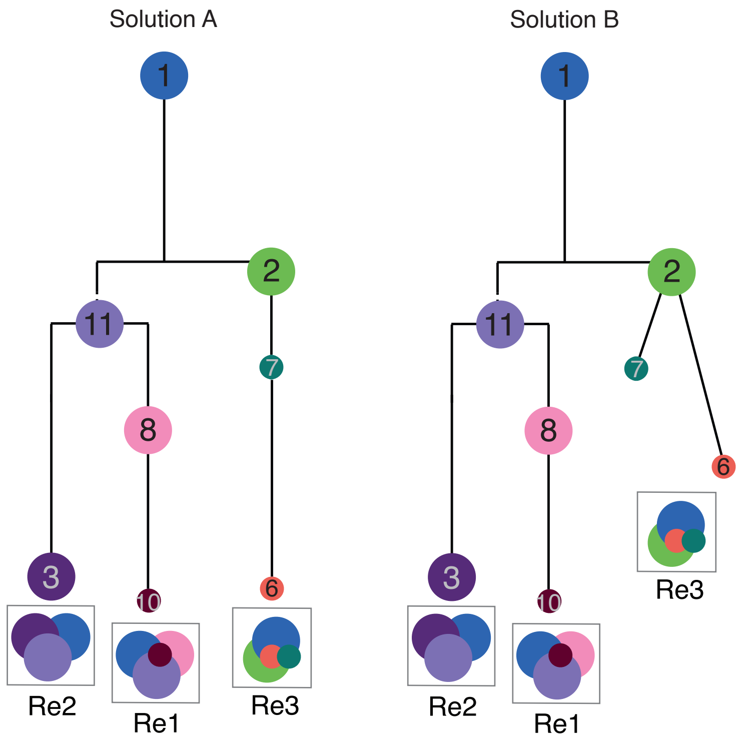


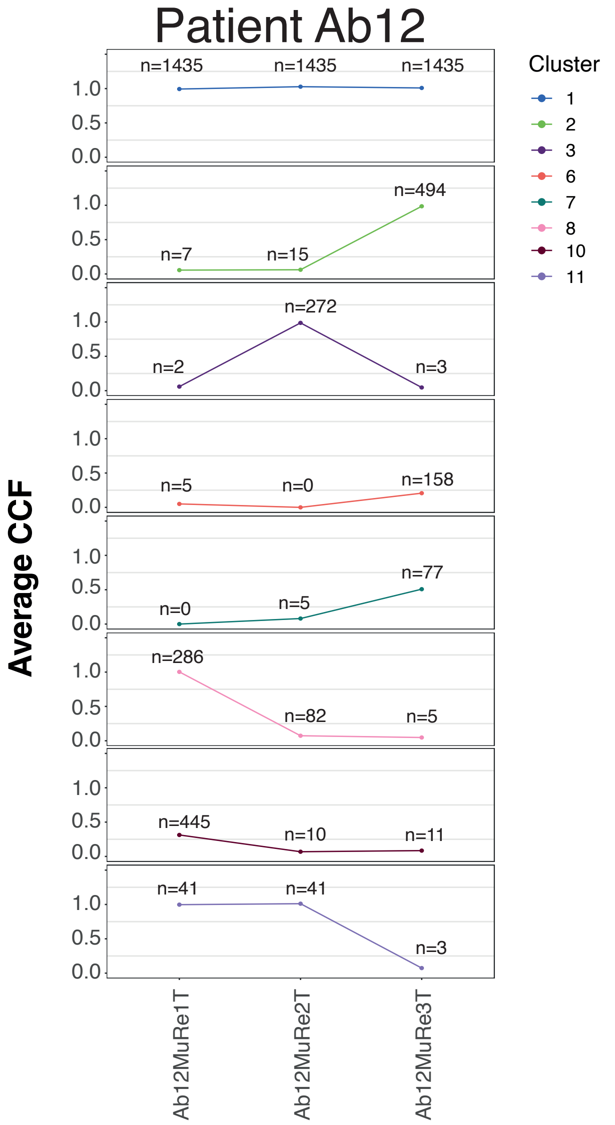


Supplementary Figure 21

**Supplementary Figure 21. DPClust Subclonal Reconstruction of Patients Ab17 and Ab12.** Treeomics-based phylogenetic trees (left) can be validated and refined by DPClust subclonal reconstruction. Diagnostic specimens (Dx), metastatic lesions (Met) and multiregion samples (Regions/Re) are shown. The average cancer cell fraction (CCF) per sample per cluster is shown (right dot plots). The CCF is the fraction of tumor cells carrying the mutation, where the clonal mutations appear in a cluster >0.9, as they are found more than 90% of tumor cells, and the subpopulation consists of tumor cells below 0.9. The CCF is based on the variant allele frequency (VAF), tumor purity and local copy number changes. Dot plots are annotated with the number of mutations assigned to each cluster in each sample. The color of each circle represents a distinct clone population (or cluster). Clones between samples are considered shared if the CCF of the clones is greater than 5% and the number of mutations in the cluster is greater than 5% of the total number of mutations assigned to the cluster. There must also be at least 5 mutations in each clone. DPClust produces two plausible trees for patient Ab12, the linear model was chosen for Figure 3B. Branch lengths are proportional to Treeomics mutation assignments, except when DPClust further resolves subclonal populations. In these cases, DPClust mutation assignments are used.

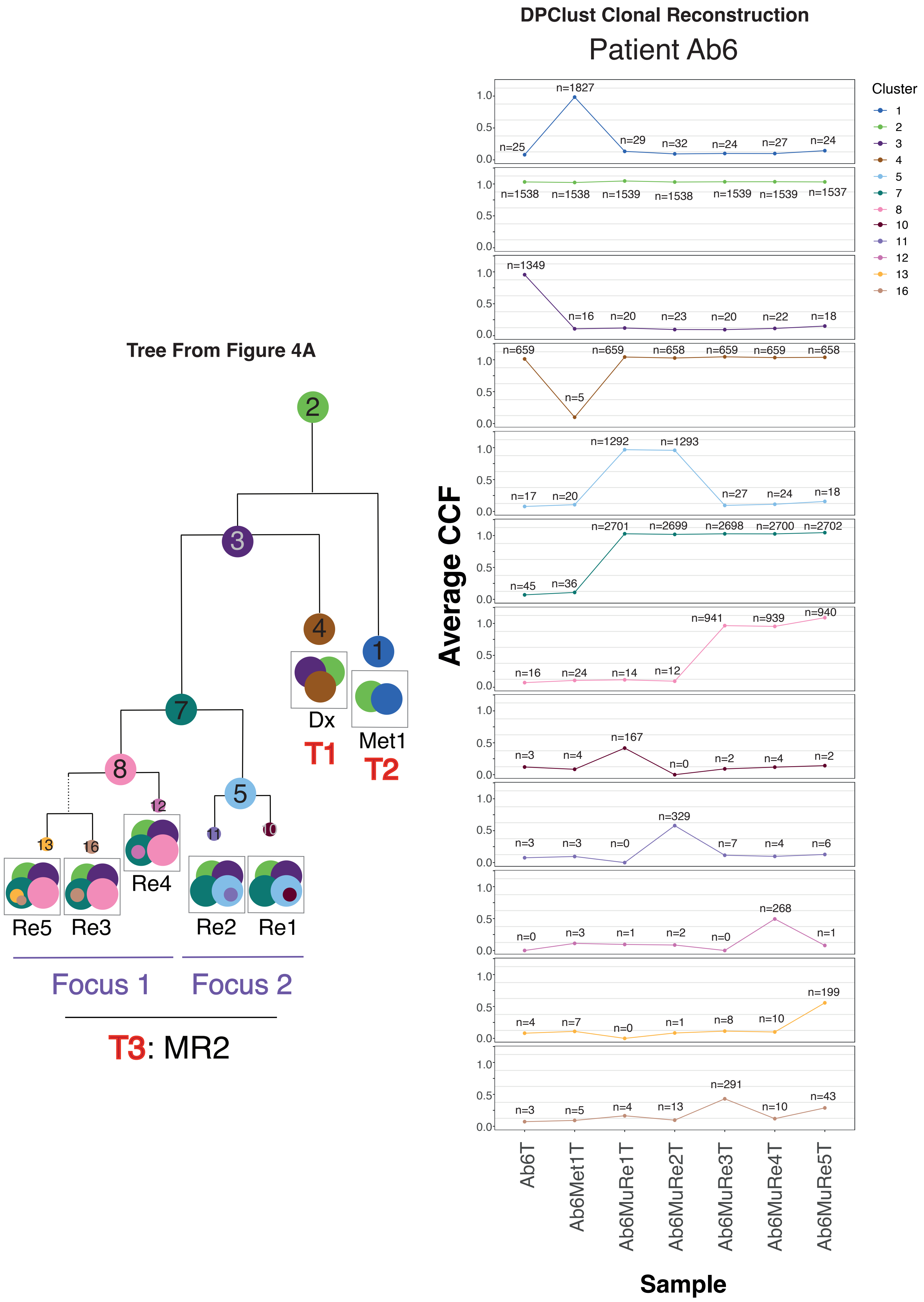
DPClust Clonal Reconstruction
Patient Ab6
Tree From Figure 4A
Dx
T1
Met1
T2
Re5
Re3
Re4
Re2
Re1
Focus 1
Focus 2
T3: MR2
Average CCF
Sample
Cluster
Ab6T
Ab6Met1T
Ab6MuRe1T
Ab6MuRe2T
Ab6MuRe3T
Ab6MuRe4T
Ab6MuRe5T

**Supplementary Figure 22. DPClust Subclonal Reconstruction of Patient Ab6.** Treeomics-based phylogenetic trees (left) can be validated and refined by DPClust subclonal reconstruction. Diagnostic specimens (Dx), metastatic lesions (Met) and multiregion samples (Regions/Re) are shown. The average cancer cell fraction (CCF) per sample per cluster is shown (right dot plots). The CCF is the fraction of tumor cells carrying the mutation, where the clonal mutations appear in a cluster >0.9, as they are found more than 90% of tumor cells, and the subpopulation consists of tumor cells below 0.9. The CCF is based on the variant allele frequency (VAF), tumor purity and local copy number changes. Dot plots are annotated with the number of mutations assigned to each cluster in each sample. The color of each circle represents a distinct clone population (or cluster). Clones between samples are considered shared if the CCF of the clones is greater than 5% and the number of mutations in the cluster is greater than 5% of the total number of mutations assigned to the cluster. There must also be at least 5 mutations in each clone. Branch lengths are proportional to Treeomics mutation assignments, except when DPClust further resolves subclonal populations. In these cases, DPClust mutation assignments are used.

## Tree from Figure 4B

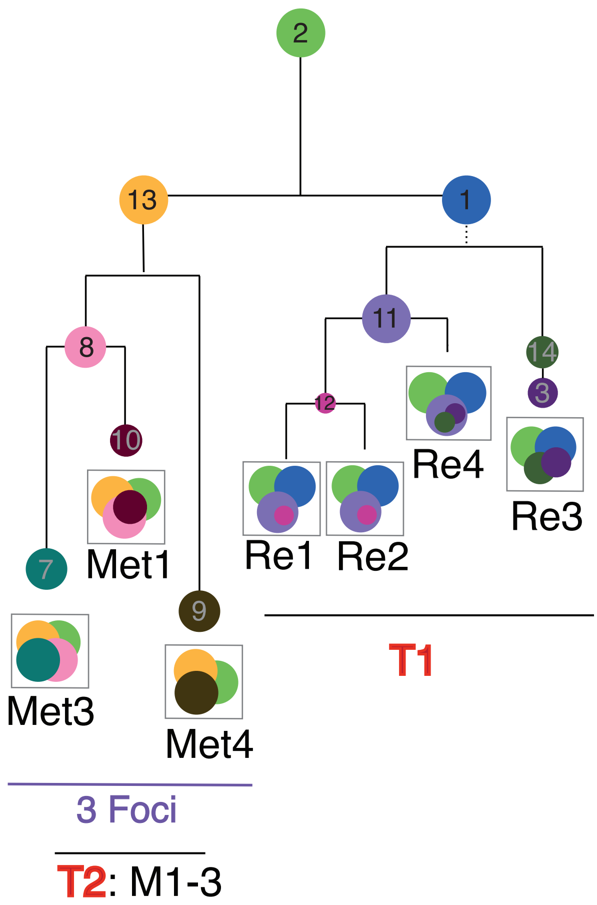


## DPClust Clonal Reconstruction

## Patient Ab11

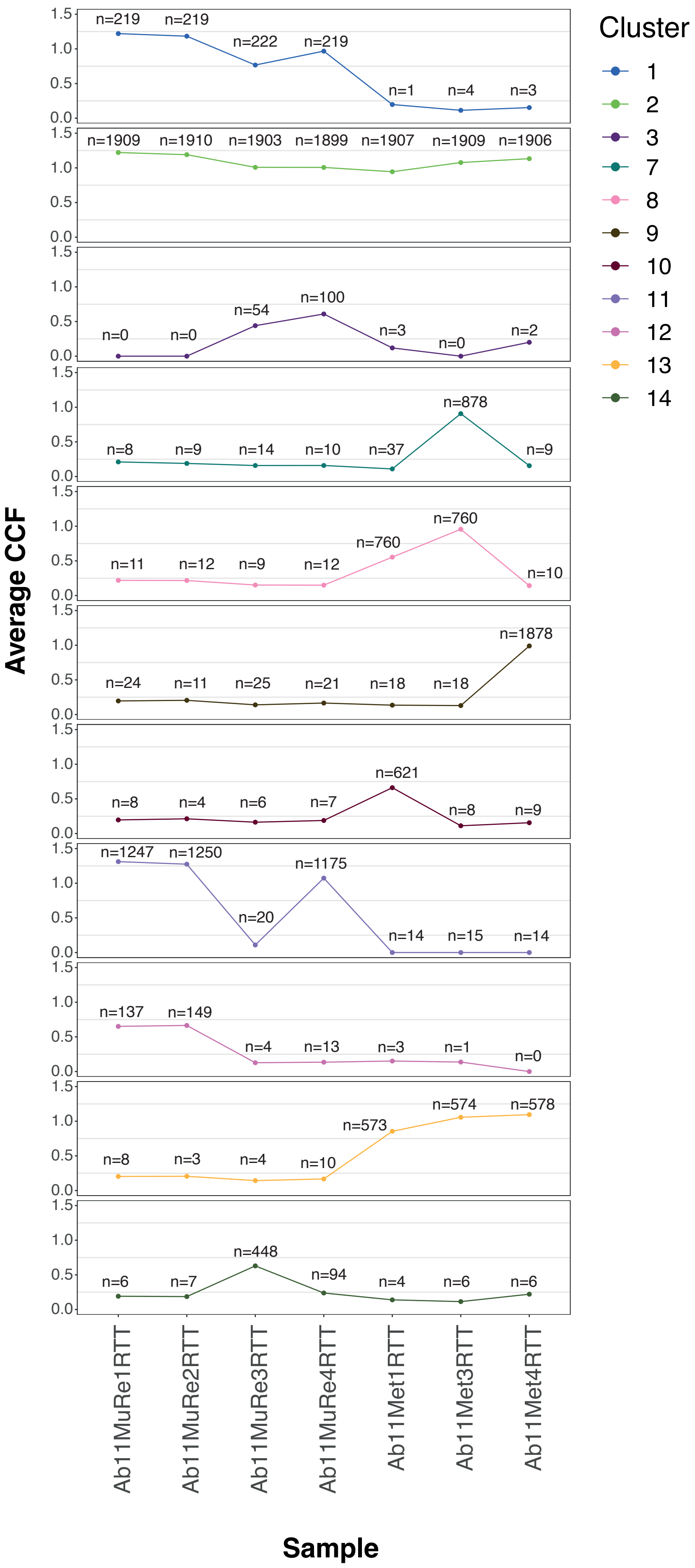


Supplementary Figure 23

**Supplementary Figure 23. DPClust Subclonal Reconstruction of Patient Ab11.** Treeomics-based phylogenetic trees (left) can be validated and refined by DPClust subclonal reconstruction. Diagnostic specimens (Dx), metastatic lesions (Met) and multiregion samples (Regions/Re) are shown. The average cancer cell fraction (CCF) per sample per cluster is shown (right dot plots). The CCF is the fraction of tumor cells carrying the mutation, where the clonal mutations appear in a cluster >0.9, as they are found more than 90% of tumor cells, and the subpopulation consists of tumor cells below 0.9. The CCF is based on the variant allele frequency (VAF), tumor purity and local copy number changes. Dot plots are annotated with the number of mutations assigned to each cluster in each sample. The color of each circle represents a distinct clone population (or cluster). Clones between samples are considered shared if the CCF of the clones is greater than 5% and the number of mutations in the cluster is greater than 5% of the total number of mutations assigned to the cluster. There must also be at least 5 mutations in each clone. Branch lengths are proportional to Treeomics mutation assignments, except when DPClust further resolves subclonal populations. In these cases, DPClust mutation assignments are used.

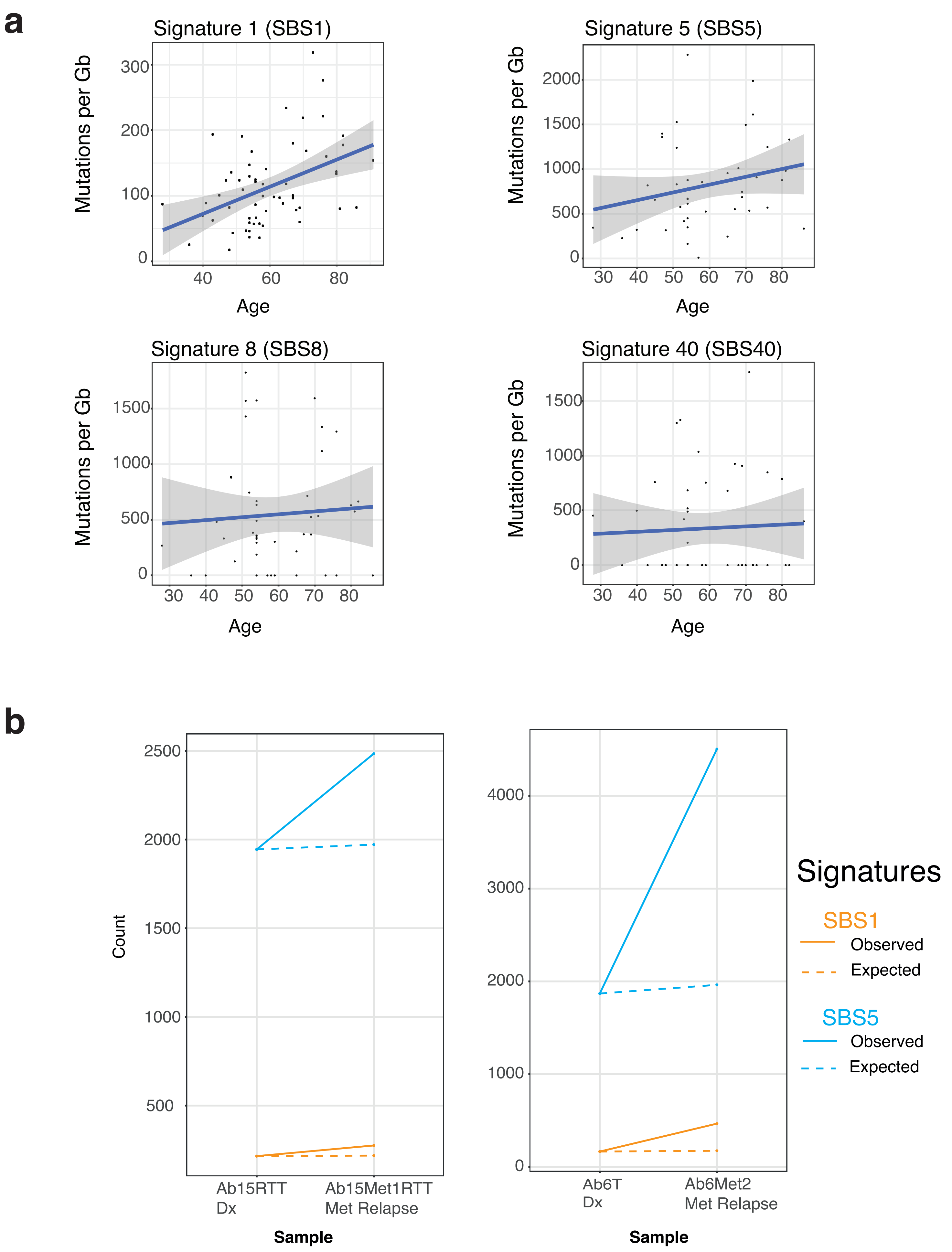


Supplementary Figure 24

**Supplementary Figure 24. Single Base Substitution (SBS) 1 is "clock-like" in LMS and increases with age. (A)** The mutation rates (mutation/Gb/year) are plotted against the age of the patient for SBS1, SBS5, SBS8 and SBS40. The strongest correlation between mutation rate and age are seen for SBS1. SBS1 reflect the number of mitotic cell divisions, which is proportional to the chronological age of LMS patients. Ribbons represent the 95% confidence interval of the fitted lines. **(B)** Given the calculated SBS1 and SBS5 mutation rates (mutation/Gb/year) for two representative patients (Ab6 and Ab15), the expected number SBS1 and SBS5 mutations was determined for the relapse samples. These were compared to the NMF-extracted SBS1 and SBS5 numbers from the relapse samples (observed). Only for SBS1, not SBS5 (or any other signature), was the expected and observed mutation accumulation similar.

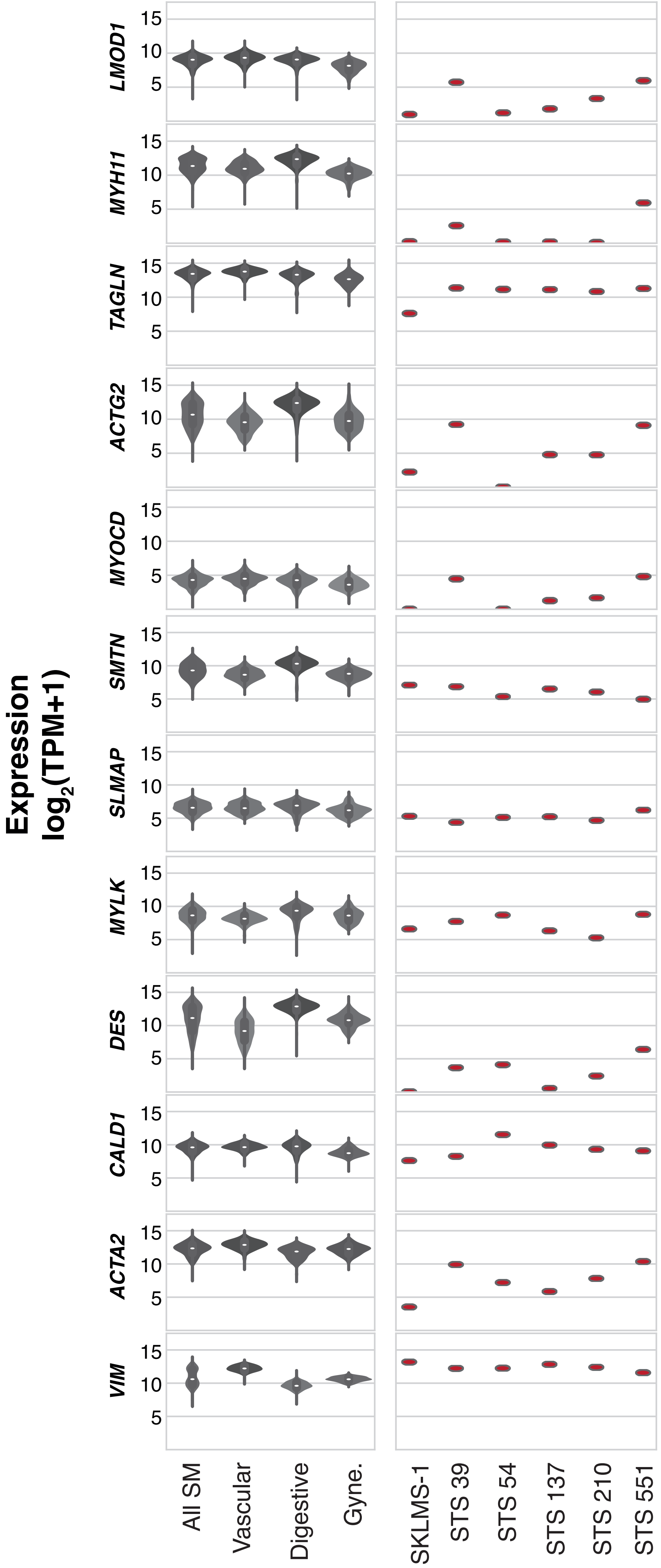


Supplementary Figure 25

**Supplementary Figure 25. Smooth Muscle (SM) Gene Expression in Patient-Derived Cell Lines.** Plots show the expression (transcripts per million, TPM) of smooth muscle genes in normal tissue SM (left): vascular (n=110), digestive (n=119) and gynecological (Gyn., n=42) tissues. SM expression for SKLMS-1 and patient-derived cell lines are shown on the right (each point represents a single cell line). The boxes represent the 25th and 75th percentile (bottom and top of box), and median value (horizontal band). The whiskers indicate the variability outside the upper and lower quartiles.

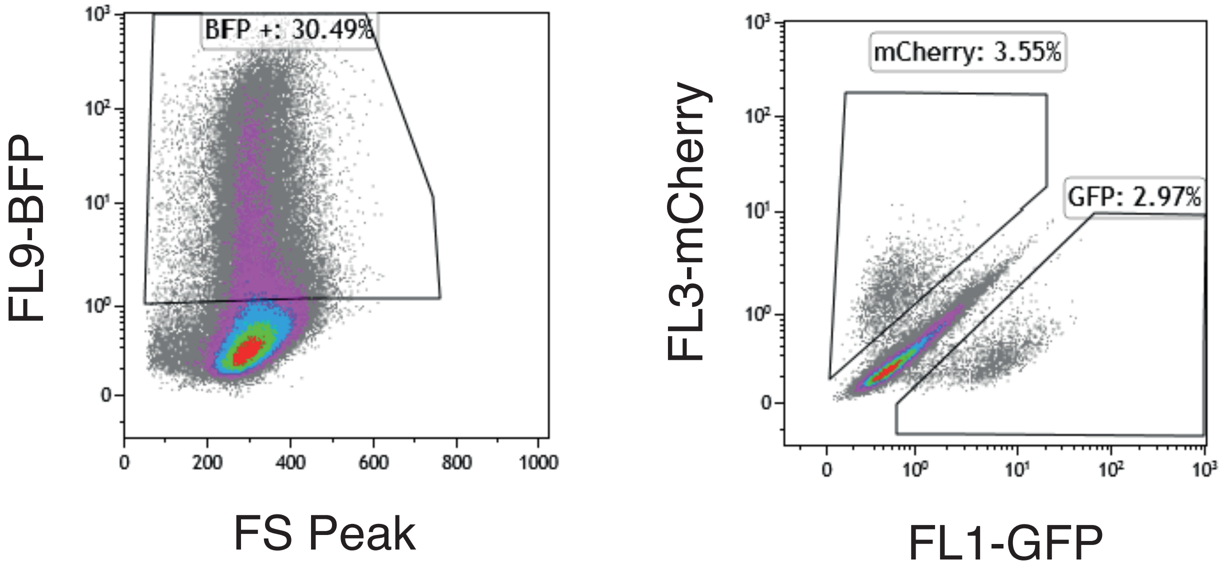


**Supplemental Figure 26. Gating strategy for Traffic Light Reporter (TLR) assay.** Blue Fluorescent Protein (BFP) positive cells were gated as the signal (Fluorescecnce channel (FL) 9 parameter: 405nm laser, 450/40 bandpass filter detector) above the untransfected control. From the BFP positive cells, Green Fluorescent Protein (GFP) (FL1 parameter: 488nm laser, 525/40 bandpass filter detector) and mCherry (FL3 parameter: 561nm laser, 620/30 bandpass filter detector) signals were plotted and gates were created to identify the BFP+ GFP+ mCherry- or BFP+ GFP- mCherry+ cells. FS refers to the forward scatter.

# Patient Ab11

MuRe1

Ploidy: 3.32, aberrant cell fraction: 31%, goodness of fit: 84.3%

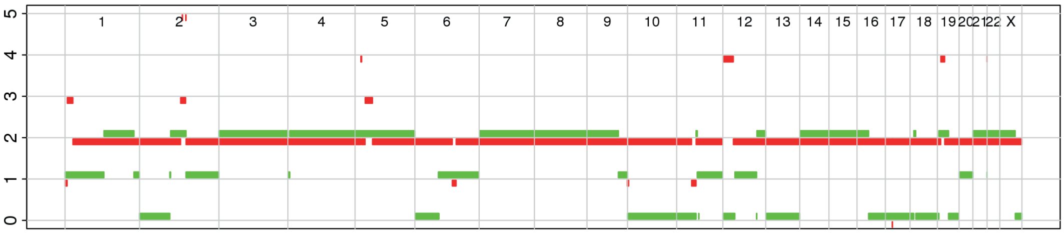


MuRe2

Ploidy: 3.29, aberrant cell fraction: 36%, goodness of fit: 84.6%

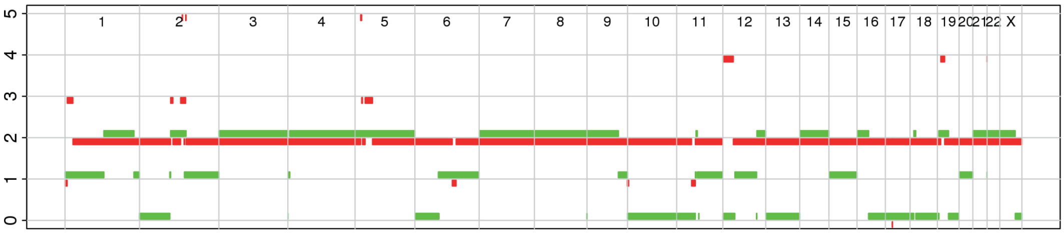


MuRe3

Ploidy: 1.89, aberrant cell fraction: 35%, goodness of fit: 68.0%

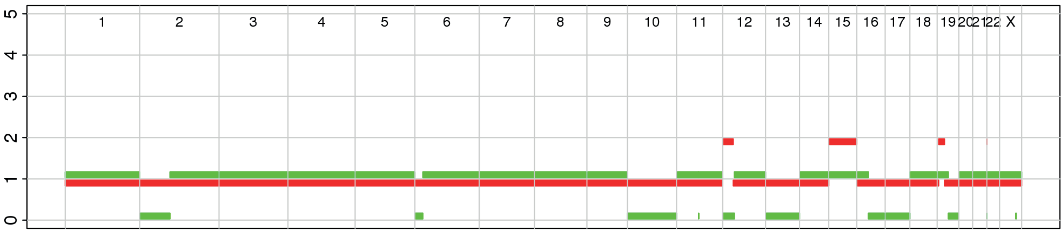


MuRe4

Ploidy: 1.70, aberrant cell fraction: 32%, goodness of fit: 57.2%

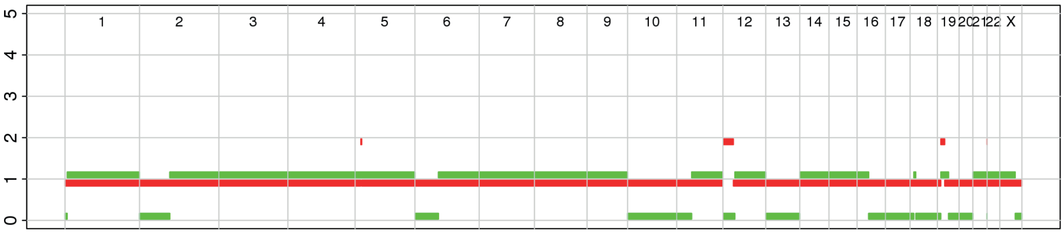


Met1

Ploidy: 1.81, aberrant cell fraction: 39%, goodness of fit: 58.7%

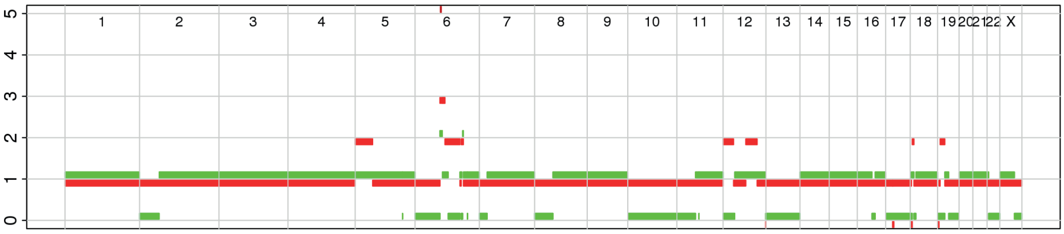


Met3

Ploidy: 3.60, aberrant cell fraction: 75%, goodness of fit: 82.7%

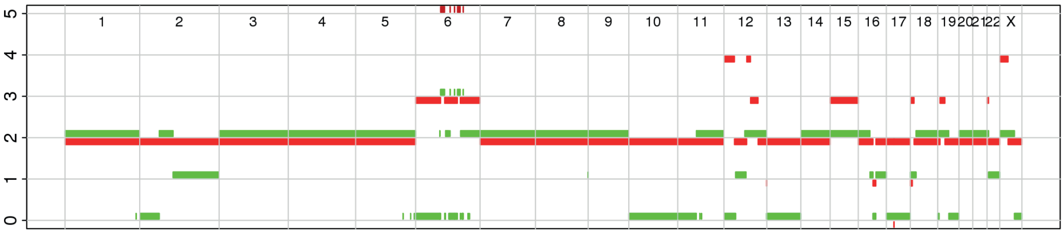


Met4

Ploidy: 3.35, aberrant cell fraction: 47%, goodness of fit: 79.8%

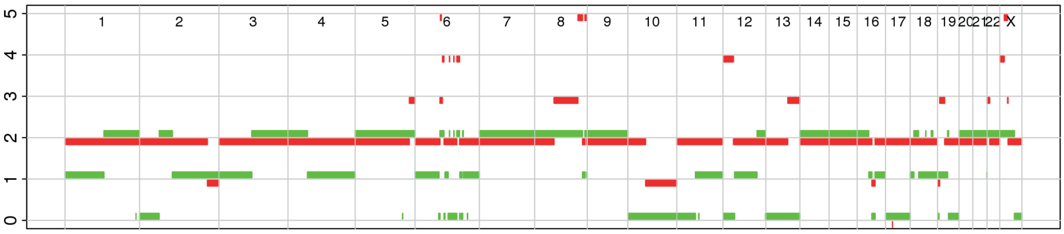


Supplementary Figure 27

**Supplementary Figure 27. Quality Control for Patient Ab11.** Battenberg derived copy number plots are provided for patient Ab11. Based on Treeomics and DPClust reconstructions, multiregion (MuRe) 3, MuRe4 and Met1 likely have missed a genome doubling event (see Methods).
