# Supplementary material for: Lineage-defined leiomyosarcoma subtypes emerge years before diagnosis and determine patient survival
Source: Nat Commun. 2021 Jul 23;12:4496. doi: 10.1038/s41467-021-24677-6 (PMC8302638; doi:10.1038/s41467-021-24677-6)
Supplement: Supplementary file 3 — Description of Additional Supplementary Files [file 41467_2021_24677_MOESM3_ESM.pdf]

Description of Additional Supplementary Files

File Name: Supplementary Data 1
Description: This table shows the most mutated genes by point mutations (per genome and per patient). Gene-level mutations must occur in at least two patients and at least three genomes to be included.

File Name: Supplementary Data 2
Description: This table shows the most mutated genes by insertion/deletions (indels) (per genome and per patient). Indels must occur in at least two patients.

File Name: Supplementary Data 3
Description: This table shows the most recurrent structural variant (SV) events (per patient). At least one side of the SV must be genic to be considered. The SV must occur in at least two patients.

File Name: Supplementary Data 4
Description: This table shows allele-specific copy number (major and minor copy number) and copy number state for key LMS genes.

File Name: Supplementary Data 5
Description: This table shows the approximate loci of C>T and/or C>G clustered mutations which occurred at TpCpN trinucleotide. These are considered kataegis events.

File Name: Supplementary Data 6
Description: This table shows the loci of chromothripsis events.

File Name: Supplementary Data 7
Description: This table shows the subtype and genomic mutation burdens of LMS patients' genomes.

File Name: Supplementary Data 8
Description: This table outlines the pathologic information on primary LMS cell lines derived from tumors in the Toronto Sarcoma Program.

File Name: Supplementary Data 9
Description: This table outlines the pathologic information on primary Undifferentiated Pleiomorphic Sarcoma (UPS) cell lines derived from tumors in the Toronto Sarcoma Program.

File Name: Supplementary Data 10
Description: This table shows the demographics and clinical characteristics of LMS patients included in this study.

File Name: Supplementary Data 11
Description: This table shows the quality and QC metrics of RNA used in this study. RNA area and integrity number (RIN) were calculated by 2100 Bioanalyzer Instrument (Agilent Technologies, Inc).
