# Supplementary material for: Lineage-defined leiomyosarcoma subtypes emerge years before diagnosis and determine patient survival
Source: Nat Commun. 2021 Jul 23;12:4496. doi: 10.1038/s41467-021-24677-6 (PMC8302638; doi:10.1038/s41467-021-24677-6)
Supplement: Supplementary file 5 — Reporting Summary [file 41467_2021_24677_MOESM5_ESM.pdf]

nature research

Corresponding author(s): Adam Shlien and Rebecca A. Gladdy

Last updated by author(s): Jun 17, 2021

Reporting Summary

Nature Research wishes to improve the reproducibility of the work that we publish. This form provides structure for consistency and transparency in reporting. For further information on Nature Research policies, see our Editorial Policies and the Editorial Policy Checklist.

Statistics

For all statistical analyses, confirm that the following items are present in the figure legend, table legend, main text, or Methods section.

n/a	Confirmed	
☐	☒	The exact sample size (*n*) for each experimental group/condition, given as a discrete number and unit of measurement
☒	☐	A statement on whether measurements were taken from distinct samples or whether the same sample was measured repeatedly
☐	☒	The statistical test(s) used AND whether they are one- or two-sided<br>*Only common tests should be described solely by name; describe more complex techniques in the Methods section.*
☐	☒	A description of all covariates tested
☒	☐	A description of any assumptions or corrections, such as tests of normality and adjustment for multiple comparisons
☐	☒	A full description of the statistical parameters including central tendency (e.g. means) or other basic estimates (e.g. regression coefficient) AND variation (e.g. standard deviation) or associated estimates of uncertainty (e.g. confidence intervals)
☐	☒	For null hypothesis testing, the test statistic (e.g. *F*, *t*, *r*) with confidence intervals, effect sizes, degrees of freedom and *P* value noted<br>*Give P values as exact values whenever suitable.*
☒	☐	For Bayesian analysis, information on the choice of priors and Markov chain Monte Carlo settings
☒	☐	For hierarchical and complex designs, identification of the appropriate level for tests and full reporting of outcomes
☐	☒	Estimates of effect sizes (e.g. Cohen's *d*, Pearson's *r*), indicating how they were calculated

Our web collection on statistics for biologists contains articles on many of the points above.

Software and code

Policy information about availability of computer code

Data collection	No software was used for data collection.
Data analysis	For our analyses, paired-end FASTQ files were aligned to the human genome (hg19/GRCh37) using BWA-MEM (v.0.7.8), Picard MarkDuplicates (v.1.108) was used to mark PCR duplicates. Indel realignment and base quality scores were recalibrated using the Genome Analysis Toolkit (v.2.8.1). RNA-seq reads were aligned on human genome using STAR (v.2.4.2). We obtained gene expression counts (TPM) by running samples through the toil-RNASeq pipeline (which includes cutadap v.1.9, STAR v.2.4.2a and RSEM v.1.2.25). Normalization was performed using Trimmed Mean of M-value (TMM) method in EdgeR (v.3.34.0) on genes with at least 1 read per million bases in at least 3 samples. Normalized data were then log transformed and the removeBatchEffects function from the limma package (v.3.48.0) in R was used to remove any source of variability from different experiments. We detected somatic mutations using established tools (MuTect2 (part of GATK v.3.8)47 and Delly v.0.7.1)), and used custom filters, as described previously (PMID: 30166462). Depth-based and allele-specific copy-number were detected from whole-genome sequences using BIC-seq (v.1.2.1) and Battenberg (v.3.2.2), respectively. To investigate mutational heterogeneity and evolutionary dynamics in LMS, we used Treeomics (v.1.7.12). Custom code described in this study is available at github.com/shlienlab.

For manuscripts utilizing custom algorithms or software that are central to the research but not yet described in published literature, software must be made available to editors and reviewers. We strongly encourage code deposition in a community repository (e.g. GitHub). See the Nature Research guidelines for submitting code & software for further information.

Data

Policy information about availability of data

All manuscripts must include a data availability statement. This statement should provide the following information, where applicable:

- Accession codes, unique identifiers, or web links for publicly available datasets
- A list of figures that have associated raw data
- A description of any restrictions on data availability

Raw sequencing data generated in this study have been deposited in the European Genome-phenome Archive (EGA) with the accession numbers EGAS00001004783 (RNA-seq) [https://ega-archive.org/studies/EGAS00001004783] and EGAS00001005341 (WGS) [https://ega-archive.org/studies/EGAS00001005341]. Published LMS samples that were sequenced as part of TCGA were downloaded from the NIH Genomic Data Commons (GDC) Data Portal [http://gdc.nci.nih.gov/]. GTeX RNA-sequencing data was downloaded from the GTeX data portal [https://www.gtexportal.org/home/]. Source data are provided with this paper.

Field-specific reporting

Please select the one below that is the best fit for your research. If you are not sure, read the appropriate sections before making your selection.

☒ Life sciences ☐ Behavioural & social sciences ☐ Ecological, evolutionary & environmental sciences

For a reference copy of the document with all sections, see nature.com/documents/nr-reporting-summary-flat.pdf

Life sciences study design

All studies must disclose on these points even when the disclosure is negative.

Sample size	44 LMS tumors from 34 patients and matched-blood samples were collected at Mount Sinai Hospital and the University Health Network (UHN) in Toronto, Canada. An additional 80 samples were obtained from the Cancer Genome Atlas (TCGA). Sample sizes were chosen based on tumor and matched normal availability. The sample size is sufficient because, despite being a rare tumor, this is the largest amalgamation of LMS NGS data to-date.
Data exclusions	1 TCGA sample was excluded due to the presence of a KIT variant. This variant changes the diagnosis from a LMS to a GIST.
Replication	For our functional analysis, LMS cell lines were treated with drugs at 24-point concentrations in three independent experiments. All replications were successful.
Randomization	Randomization is not relevant to this study as participants were not allocated to experimental groups. For functional studies, we did not randomize the cell lines.
Blinding	Blinding is not relevant to this study as participants were not allocated to experimental groups. Blinding was not performed for the cell line inhibitor experiments. Inhibitor studies for LMS and UPS cell lines were performed independently.

Reporting for specific materials, systems and methods

We require information from authors about some types of materials, experimental systems and methods used in many studies. Here, indicate whether each material, system or method listed is relevant to your study. If you are not sure if a list item applies to your research, read the appropriate section before selecting a response.

Materials & experimental systems

n/a	Involved in the study	
☒	☐	Antibodies
☐	☒	Eukaryotic cell lines
☒	☐	Palaeontology and archaeology
☒	☐	Animals and other organisms
☐	☒	Human research participants
☒	☐	Clinical data
☒	☐	Dual use research of concern

Methods

n/a	Involved in the study	
☒	☐	ChIP-seq
☐	☒	Flow cytometry
☒	☐	MRI-based neuroimaging

Eukaryotic cell lines

Policy information about cell lines

Cell line source(s)	Primary patient-derived LMS cell lines (STS39, STS54, STS137, STS210, STS551) were established from confirmed surgical specimens in accordance with institutional research ethics at Mount Sinai Hospital in Toronto, Canada. All cases were reviewed by a dedicated sarcoma pathologist. SKLMS-1, SKUT1, and SKUT-1B cell lines were obtained from ATCC (Manassas, VA, USA). RPEΔp53 and RPEΔp53ΔBRCA1 were kind gift of D. Durocher. Hs-789.Sk was obtained from ATCC (Manassas, VA, USA)
Authentication	All cell lines are routinely authenticated by STR-analysis at The Centre for Applied Genomics (TCAG) SickKids in Toronto.
Mycoplasma contamination	All cell lines tested negative for mycoplasma contamination.
Commonly misidentified lines (See ICLAC register)	No misidentified cell lines were used in this study.

Human research participants

Policy information about studies involving human research participants

Population characteristics	Patients diagnosed with a leiomyosarcoma at Mount Sinai Hospital and the University Hospital Network in Toronto are eligible for this study. The patients' clinical features and demographics were typical of LMS: the average age at diagnosis was 56.5 years (28 to 87 yrs.); the male to female ratio was 1:2.6 (abdominal/extremity only).
Recruitment	Patients were recruited and consented if they have a biopsy-proven leiomyosarcoma diagnosis and are receiving surgical treatment at the Mount Sinai/UHN. Tissues used in the study were selected based on pathologic assessment ensuring adequate viability of tumor for NGS. Therefore, self-selection bias is not plausible here.
Ethics oversight	Leiomyosarcoma tumor and matched-blood and/or adjacent normal tissue samples were collected at Mount Sinai Hospital and the University Health Network (UHN) in Toronto, Canada in accordance with each institutions' Research Ethical Board (REB) guidelines. Written informed consent was obtained from all patients enrolled in the LMS Genomics Program, including permission to publish indirect identifiers.

Note that full information on the approval of the study protocol must also be provided in the manuscript.

Flow Cytometry

Plots

Confirm that:

- [x] The axis labels state the marker and fluorochrome used (e.g. CD4-FITC).
- [x] The axis scales are clearly visible. Include numbers along axes only for bottom left plot of group (a 'group' is an analysis of identical markers).
- [x] All plots are contour plots with outliers or pseudocolor plots.
- [x] A numerical value for number of cells or percentage (with statistics) is provided.

Methodology

Sample preparation	LMS-TLR cell lines were nucleofected with with 5 µg of pCVL.SFFV.d14GFP.Ef1a.HA.NLS.Sce(opt).T2A.TagBFP plasmid DNA (DD4886, Addgene #32627) in 100 µL of electroporation buffer (25 mM Na2HPO4 pH 7.75, 2.5 mM KCl, 11 mM MgCl2), using program T23 on a Nucleofector 2b (Lonza). After 96 h, GFP and mCherry fluorescence was assessed in BFP-positive cells
Instrument	Gallios flow cytometer (Beckman Coulter, Miami, FL, USA) equipped with 4 lasers (405nm, 488nm, 561nm, 633nm) running Gallios acquisition software
Software	Software for collection and analyzing: analysis software is Kaluza v2.1.2 (Beckman Coulter)
Cell population abundance	BFP positive cells - 30-60%
Gating strategy	As the first step we distinguished populations of cells based on their forward and side scatter properties. A BFP positive cells were gated as signal (FL9 parameter: 405nm laser, 450/40 bandpass filter detector) above the untransfected control. From the BFP positive cells, GFP (FL1 parameter: 488nm laser, 525/40 bandpass filter detector) and mCherry (FL3 parameter: 561nm laser, 620/30 bandpass filter detector) signals were plotted and gates were created to identify the BFP+ GFP+ mCherry- or BFP+ GFP- mCherry+ cells.

- [x] Tick this box to confirm that a figure exemplifying the gating strategy is provided in the Supplementary Information.
